# Novel Hybrid Benzoazacrown Ligand as a Chelator for Copper and Lead Cations: What Difference Does Pyridine Make

**DOI:** 10.3390/molecules27103115

**Published:** 2022-05-12

**Authors:** Bayirta V. Egorova, Lyubov S. Zamurueva, Anastasia D. Zubenko, Anna V. Pashanova, Artem A. Mitrofanov, Anna B. Priselkova, Yuri V. Fedorov, Alexander L. Trigub, Olga A. Fedorova, Stepan N. Kalmykov

**Affiliations:** 1 Chemistry Department, Lomonosov Moscow State University, Leninskie Gory, 1, 119991 Moscow, Russia; lyubovzam@mail.ru (L.S.Z.); mitrofjr@gmail.com (A.A.M.); stepan@radio.chem.msu.ru (S.N.K.); 2 A. N. Nesmeyanov Institute of Organoelement Compounds of the Russian Academy of Sciences, Vavilova, 28, GSP-1, 119991 Moscow, Russia; nastya.mutasova@yandex.ru (A.D.Z.); anya-pash107@yandex.ru (A.V.P.); fedorov@ineos.ac.ru (Y.V.F.); 3 Higher Chemical College, Mendeleev University of Chemistry and Technology of Russia, Miusskaya Sqr., 9, 125047 Moscow, Russia; 4 Skobeltsyn Institute of Nuclear Physics, Lomonosov Moscow State University, Leninskie Gory, 1, 119991 Moscow, Russia; apriselkova@mail.ru; 5 National Research Center “Kurchatov Institute”, Akademika Kurchatova Sqr., 1, 123098 Moscow, Russia; alexander.trigub@gmail.com

**Keywords:** azacrown ether, stability of complexes, radiopharmaceutical, copper-64, lead-212, list three to ten

## Abstract

A synthetic procedure for the synthesis of azacrown ethers with a combination of pendant arms has been developed and the synthesized ligand, characterized by various techniques, was studied. The prepared benzoazacrown ether with hybrid pendant arms and its complexes with copper and lead cations were studied in terms of biomedical applications. Similarly to a fully acetate analog, the new one binds both cations with close stability constants, despite the decrease in both constants. The calculated geometry of the complexes correlate with the data from X-ray absorption and NMR spectroscopy. Coordination of both cations differs due to the difference between the ionic radii. However, these chelation modes provide effective shielding of cations in both cases, that was shown by the stability of their complexes in the biologically relevant media towards transchelation and transmetallation.

## 1. Introduction

**Figure 1:**
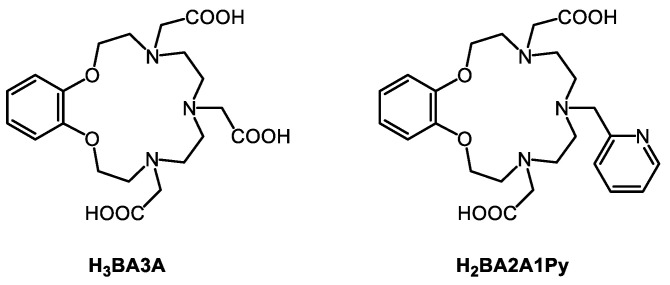
Ligands H_3_BA3A and H_2_**BA2A1Py**.

Given the growing importance of nuclear medicine and the extending abilities of obtaining medically relevant radiometals, the design of functional polyazamacrocyclic ligands able to coordinate cations with suitable features has been attracting increased attention during the last decade. In the frame of systematic variation and the search for the best matching pairs between medical radiometals and chelators, we have synthesized those substituted with one type of arm and arm-free pyridine bisamide azacrown and benzoazacrown ethers [1,2,3,4,5,6]. We have shown that azacrown compounds with acetic arms are effective for binding bismuth [4,5], lead [7] and zinc [8] cations, regarding the nuclear medicine application of their radioisotopes. However, these ligands, including H_3_BA3A (Figure 1)—the closest analog of the ligand presented here, were unable to strongly chelate Cu^2+^. Given Cu^2+^ is a transition metal cation, preferring softer N-donors along with O-donor atoms, it can be effectively bound by aromatic nitrogen that is known to be less basic than aliphatic amines. Moreover, it has been recently shown that H_4_DOTA derivatives with a pyridine arm can significantly improve copper binding [9].

To the best of our knowledge, protonation and complexation constants of such hybrid ligands with the simultaneous presence of acetic and pyridyl pendant groups have not been described in the literature yet. There are examples of substitutions of all (two or three) acetates in azacrowns by pyridines [3,6,10,11] and it has been shown that pyridine, as a pendant arm, can significantly improve the chelation ability and kinetics of binding towards Cu^2+^ and Bi^3+^, including direct comparison with well-known H_4_DOTA [9,12,13].

In this work, we propose a new method for the synthesis of an azamacrocyclic ligand containing two types of additionally introduced coordinating acetic and pyridine groups (H_2_**BA2A1Py**, Figure 1). The Cu^2+^ and Pb^2+^ complexes with a novel ligand were studied by potentiometric methods, NMR methods, X-ray absorption spectroscopy and quantum-chemical calculations. In order to evaluate the features of radiolabeled complexes, radiolabeling with ^64^Cu and ^210^Pb isotopes has been analyzed.

## 2. Results

### 2.1. Synthesis

**Scheme 1:**
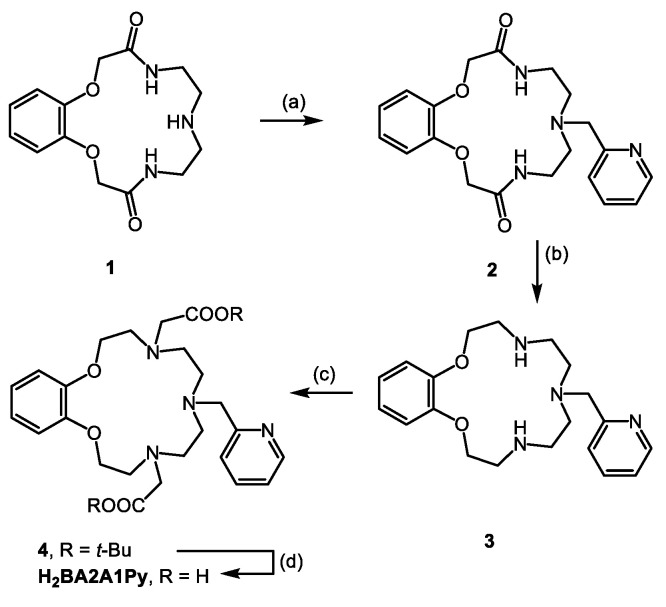
Reagents and conditions: (**a**) 2-(chloromethyl)pyridine hydrochloride, K_2_CO_3_, MeCN, reflux; (**b**) (1) BH_3_ THF, 0 °C, Ar, (2) HCl, H_2_O, reflux, (3) KOH, H_2_O, 0 °C, Ar; (**c**) *tert*-butyl bromoacetate, K_2_CO_3_, KI, MeCN, reflux; (**d**) H_2_O, reflux.

The synthesis consists of the following sequence: (1) introduction of one type of substituent in the bisamide azacrown compound; (2) reduction in amide groups; (3) introduction of another type of substituent into the obtained amino derivative (Scheme 1).

Bisamide, of 15-membered benzoazacrown compound **1**, was alkylated with chloromethylpyridine according to the method described earlier [6]. The resulting pyridyl derivative **2** was reduced using a borane–THF complex to azamacrocycle **3**, containing one side chelating substituent. In this case, the amide groups were converted into amino groups, into which the second type of substituents was further introduced. The derivative **3** was alkylated with *tert*-butyl bromoacetate in acetonitrile in the presence of a base. At the last stage, *tert*-butyl ester **4** was hydrolyzed by boiling in water without the addition of any catalysts. The prepared ligand was characterized by ^1^H and ^13^C NMR, MS, elemental analysis (ESI) and potentiometric titration.

### 2.2. Ligand Protonation

**Figure 2:**
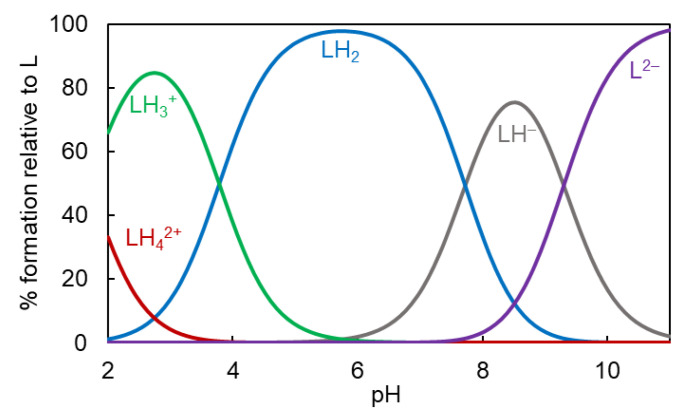
pH distribution diagrams for **BA2A1Py^2−^**.

Benzoazacrown ether H_2_**BA2A1Py** possesses several centers for proton coordination of a different nature including aliphatic and aromatic N atoms and carboxylate groups. For the determination of the protonation constants, the solution of the ligand was titrated potentiometrically with NaOH, using NaClO_4_ or KNO_3_ as background electrolytes. The obtained values of the protonation constants are given as logarithms in Table 1 and the corresponding pH distribution diagram of ligand protonated forms are shown in Figure 2.

The fully pyridine-substituted crowns described in the literature are featured by a decrease in overall basicity [3,6,10,11], but the value of the deviation of constants of the protonation steps appeared to be a function of a cumulative effect of the ligands’ structure, charge and intramolecular H-bond formation. It can be assumed that the first and the second protonation steps of **BA2A1Py**^2−^ are associated with symmetric macrocyclic amino groups as in an arm-free azacrown ether [14] while the third one is presumably related to a middle pendant arm, as in the triacetate H_3_BA3A described earlier [8]. A diprotonated form prevails in the widest range from pH 4 up to pH 7.5 (Figure 2). Furthermore, the last detected protonation constant is lower than for the triacetic ligand, correlating with the total charge of triprotonated species for H_3_BA3A and H_3_**BA2A1Py**^+^. The latter is already 1+ charged compared to the neutral H_3_BA3A and consequently possesses less affinity for protons than H_3_BA3A. Moreover, compared to acetate H_3_BA3A, the presence of pyridyl as a pendant arm decreases the overall negative charge of fully deprotonated species, that is reflected in a slight decrease in the first protonation constant. Finally, overall, a less basic character of H_2_**BA2A1Py** is observed.

**Figure 3:**
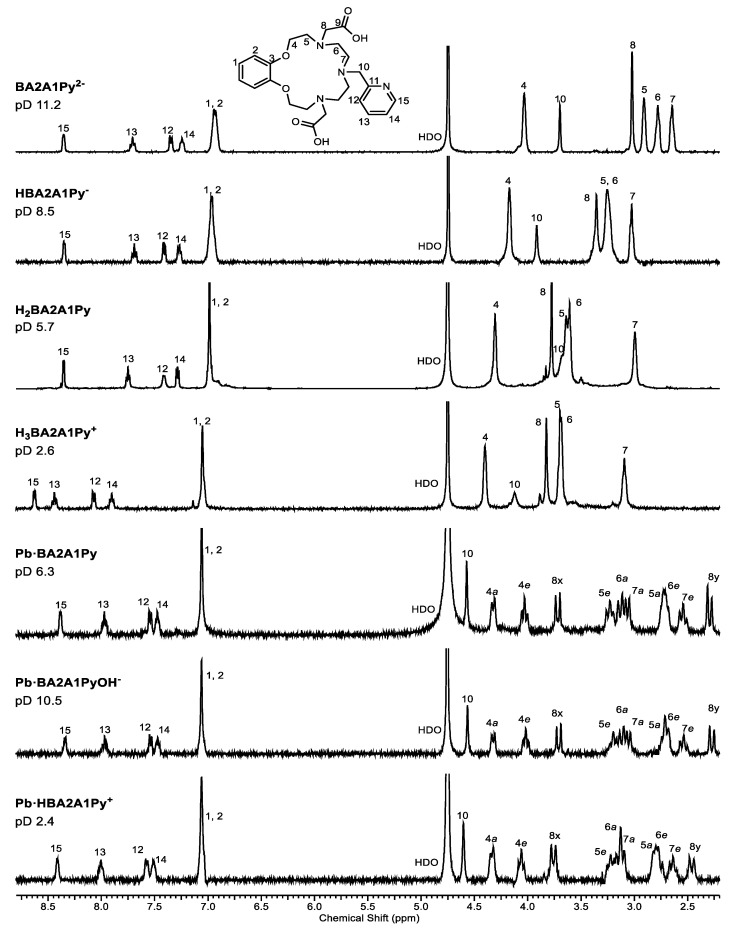
^1^H NMR spectra of ligand **H_2_BA2A1Py** and its complex with Pb^2+^ at various pD values in D_2_O.

The proposed model for ligand protonation is confirmed by the ^1^H NMR spectra of the ligand in D_2_O at various pDs, corresponding to the maximum percentage of formation of certain protonated forms in solution (Figure 2). The proton resonances of the ligand at pD 11.2 correspond to the deprotonated form **BA2A1Py^2−^** (Figure 3). At pD 8.5, the monoprotonated species H**BA2A1Py^−^** was formed and the macrocyclic proton signals in the spectrum are shifted to the downfield region, which is explained by the rapid proton exchange between the three amino groups of the ligand. Following acidification to pD 5.7, this leads to the formation of the diprotonated species H_2_**BA2A1Py**, causing the most pronounced resonance changes for macrocyclic protons H_5_, H_6_ and protons of acetate arms H_8_ (Table S1), indicating the localization of two H^+^ on the amino groups conjugated with acetate arms (Figure 3). At pD 2.6, the protons of the central arm H_10_ and the pyridine ring’s H_12–15_ exhibit a significant downfield shift (Figure 3, Table S1), which clearly indicates that the third proton is attached to the pyridine nitrogen atom, forming species H_3_**BA2A1Py^+^**.

### 2.3. Complexation Study

**Figure 4:**
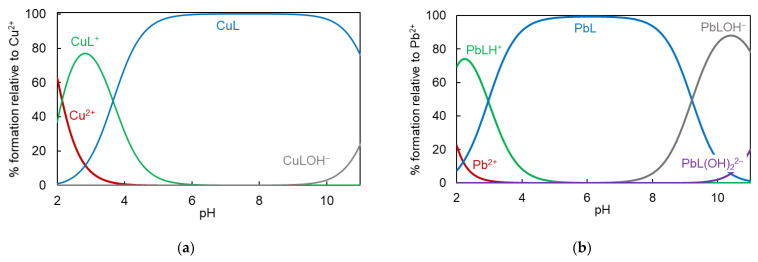
pH distribution diagrams for complexes of Cu^2+^ (**a**) and Pb^2+^ (**b**) with **BA2A1Py^2−^**.

For the determination of complex formation constants, solutions of metal perchlorates, perchloric acid and ligands with different concentrations of the components were titrated with NaOH. A potentiometric titration of ligand in the presence of equimolar concentrations of Cu^2+^ and Pb^2+^ and a two-fold excess of Cu^2+^ were performed. The complex formation constants were calculated using Hyperquad software [15]. During the determination of the stability constant values, the protonation constants of the ligand and the ionic product of water were fixed, and the hydrolysis constants of the cations were considered as known, and no attempts were made to adjust their values. The time required for equilibration in the potentiometric titration experiments was within 5 min. The obtained values are presented in the Table 1 and the corresponding pH distribution diagram of cations’ forms are shown in Figure 4. Variation of the Cu^2+^/L ratio led to the same values of logβ for LCu and HLCu: 16.24 (4) and 19.88 (2), respectively. The formation of hydroxy species with limited solubility in water hampers equilibrium constant determination. That is why, due to the formation of an insoluble hydroxide of excess of Cu^2+^ (for the experiment with Cu^2+^/L = 2/1), titration points over pH 5.5 were excluded from the calculation of complex formation constants and, consequently, the logβ of LCuOH form was not calculated from this experiment.

Both the cation formation of protonated, as well as hydroxyl complex species, is detected in the wide range of pH levels (Figure 4). Moreover, as a larger cation with a higher coordination number, Pb^2+^ forms species with one and two OH^−^ groups. The lower overall basicity of H_2_**BA2A1Py** compared to H_3_BA3A induced a consequent decrease in the logβ of complexes with **BA2A1Py^2−^**, although to a different degree: only 0.5 log for Cu^2+^, while 1.6 log for Pb^2+^ (Table 1), showing a preference towards Cu^2+^. This fact proves our suggestion about an increase in affinity for Cu^2+^ of N-donor ligands and correlates with the described CuDOTAPy in vitro stability [9].

Noteworthy, the application of NaClO_4_ and KNO_3_ as background electrolytes allowed to compare the obtained data with the literature and ensure the lack of a crucial effect on the variation of background cations to the value of the logβ(ML), at least in the pH range and at the ionic strength used. The considered conditions fairly afford the assumed biologically relevant media. The obtained values demonstrate slight changes in the formation of protonated species H_3_**BA2A1Py**^+^, H_4_**BA2A1Py**^2+^ and H**BA2A1Py**Cu^+^, apparently due to competition of Na^+^/K^+^ with H^+^ for association to carboxylic groups. However, this influence is negligible when protonation and coordination mainly occur via amino groups.

### 2.4. Complexes’ Structure Studies

**Figure 5:**
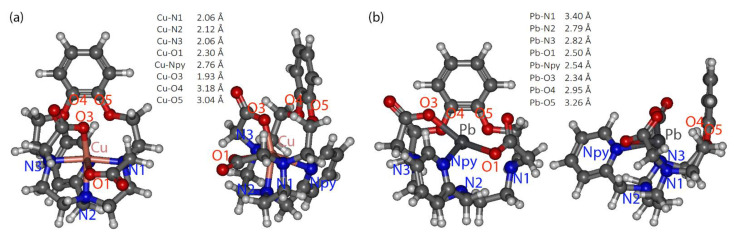
Optimized geometries of complexes of **BA2A1Py^2−^** with Cu^2+^ (**a**) and Pb^2+^ (**b**).

**Figure 6:**
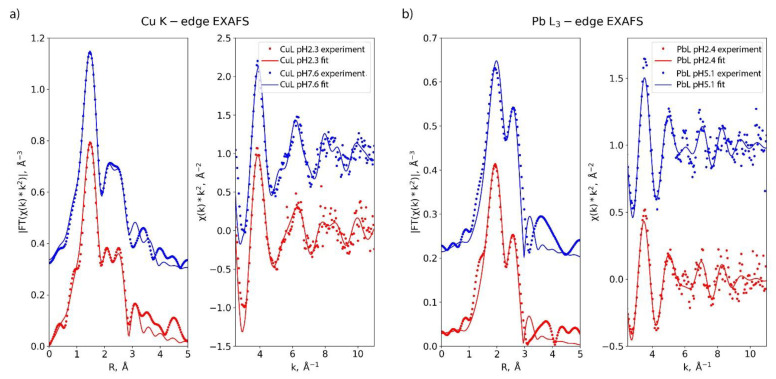
The fitted spectra for X-ray absorption of copper (**a**) and lead (**b**) complexes with H_2_**BA2A1Py** in the R- and k—spaces.

In order to evaluate the structure of these complexes in the solution, we performed DFT calculations of the geometry of both complexes (Figure 5) and compared the interatomic distances with the coordination environment obtained, by the fitting of an X-ray absorption spectra of complexes (Table 2, Figure 6).

According to the geometries obtained in both complexes a macrocyclic cavity is opened, i.e., unfolded, and depending on the ionic radius, a cation is either encapsulated inside the cavity—Cu^2+^, or coordinated above—Pb^2+^ (Figure 5).

Cu^2+^ is held by two acetates from one side of the macrocycle, and the pyridyl arm from another side, forming distorted octahedral coordination. The shortest distances (~2 Å) to three macrocyclic amino groups and one acetate group, correspond to the equatorial plane, while another acetate and pyridine group can be attributed to axial positions at 2.30 and 2.76 Å, respectively. These ranges of distances are detected in the closest distance to the Cu^2+^ shells upon EXAFS fitting: 2, 2.7 Å. Taking into account that the nearly similar atomic scattering factors of C, N and O do not allow the atoms to be distinguished in the local surrounding of the Cu atom, the total high number (N) of neighbors obtained is fair. Noteworthy is the number of atoms in the second and third coordination shells of Cu^2+^, which looks to decrease by one upon a pH increase (Table 2). Apparently it is caused by conversion of CuLH (pH2.3) to CuL (pH7.6) with a slightly higher organized arrangement in the latter structure.

In the calculated structure, a larger Pb^2+^ cation is chelated above the cavity by macrocyclic amino groups, and all pendant arms with the interatomic distances 2.3–3.4 Å to N and O donor atoms. Atoms on these ranges are found in the experimental EXAFS spectra in both cases at pH 2.4 and 5.1.

In contrast to Cu**BA2A1Py**, the number of closest neighbors increases upon conversion from the protonated form to the deprotonated form. Assumingly, it is provided by the formation of the protonated form due to the cation’s association with acetates, while in the Pb**BA2A1Py** complex, both pendant arms and the macrocycle are in the vicinity of the cation (this suggestion correlates with the NMR results described below). The latter causes a higher number of atoms at 3.3 Å. Moreover, apparently symmetric environments of Cu^2+^ vs. Pb^2+^ affects the possibility to distinguish intermediate distances to neighboring atoms, that is not the case for Pb complexes.

### 2.5. NMR Spectroscopy of PbBA2A1Py

Furthermore, the structural features of the Pb**BA2A1Py** complex in an aqueous solution were specified by NMR spectroscopy. The studies were carried out in D_2_O by comparing the ^1^H NMR spectra of the free ligand and the ligand in the presence of the Pb^2+^ cation. Changes in the ^1^H NMR spectrum observed, after the addition of the Pb^2+^ ion to the D_2_O solution of H_2_**BA2A1Py,** demonstrated geminal coupling of the methylene protons belonging to the macrocyclic fragment, as well as the pendant side arms. As a result, the eleven proton signals of the free ligand **BA2A1Py**^2−^ transform to sixteen proton signals (Figure 3). This behavior is consistent with the presence of a single complex of Pb**BA2A1Py** with a relatively rigid structure in the solution; fluxional interconversion at ambient temperatures was not observed (on the NMR time scale).

Additionally, no change in the number of peaks in the ^13^C NMR spectrum of the ligand H_2_**BA2A1Py** was found upon Pb^2+^ coordination, suggesting no chemically distinct isomers were formed (Figure S21). In the Pb**BA2A1Py** complex spectrum, the resonances of the protons of the macrocycle, benzene, and pendant arms downfield change because of the polarization effect of the metal ion (Table S1). At the same time, some signals exhibit upfield shifts. This is probably due to the diamagnetic through-space shielding effect on the chemical shifts of the protons located under the surrounding carbonyl or pyridyl units. Moreover, the large upfield shift of the acetate group signal H_8y_ indicates the shielding effect of the pyridine ring located close to them. The signal of the pyridine proton H_15_ has an insignificant downfield shift compared to the other pyridine signals, which can also confirm the close location of the acetate groups to the pyridine ring, since, in this case, the polarization effect of the metal cation is neutralized by the shielding effect of the carbonyl groups. The fact, that the methylene protons of the pyridyl substituent H_10_ appear as a singlet, can be explained by the location of this substituent in the plane of symmetry, which is perpendicular to the macrocycle and, therefore, the geminal protons H_10_ are magnetically equivalent. Considering that the diameter of the Pb^2+^ cation (2.40 Å) is larger than the size of the macrocyclic cavity (1.7–2.2 Å), it can be concluded that the Pb^2+^ cation is fixed by pyridyl and two acetate groups above the macrocyclic cavity, correlating with the calculated geometry, and the resulting complex Pb**BA2A1Py** possesses *Cs* symmetry in an aqueous solution.

When the solution of the complex of the ligand with the Pb^2+^ cation is alkalized to pD 10.5, in accordance with the data of potentiometric titration, a hydroxo- complex Pb**BA2A1PyOH^−^** is formed. The observed ^1^H NMR spectrum almost completely coincides with the spectrum of the complex Pb**BA2A1Py** (Figure 3), which means that the ligand geometry does not change. Most likely, the Pb^2+^ cation in the complex coordinates the water molecule, which in an alkaline medium turns into an OH^−^ group. Upon acidification of the solution of the complex to pD 2.4, in the ^1^H NMR spectrum, the signals of the macrocyclic protons H_5*a*_, H_6*e*_, and H_7*e*_, as well as the protons of the acetate arms H_8*y*_, exhibit a slight downfield shift relative to the spectrum of the complex Pb**BA2A1Py**, while the overall shape of the spectrum does not change (Figure 3, Table S1). It can be concluded that, upon protonation, H^+^ adds to the side amino groups of the macrocycle, while the *Cs* symmetry of the complex Pb**HBA2A1Py^+^** is retained. The latter can be caused by coordination of cations via acetates without participation of the protonated macrocycle. A similar structure of the protonated form of the complex is also characteristic of H_4_DOTA protonated intermediate complexes [16,17].

### 2.6. Ligand Lipophilicity

As one of the important features for molecules to be used as part of radiopharmaceuticals, we evaluated the lipophilicity of the molecule as the partition coefficient of **BA2A1Py^2−^** labelled by ^210^Pb between n-octanol and water. Due to the aromatic pendant arm, the obtained value *logP* = −2.11 is higher than for BA3A^3−^ (*logP* = −2.26). Of note, benzene causes a slight decrease in polarity of benzoazacrowns, compared to the smaller dipyridyl-acetic cyclononane derivative—NO1A2Py *logP* = −2.43 [12].

### 2.7. Radiolabeling of Complexes

Radiolabeled complexes were synthesized with ^64^Cu and ^210^Pb. To simulate the conditions of preparation of a highly active radiopharmaceutical we added the carrier of CuCl_2_ to reach the concentration of cations of 10 nM, as soon as 1 kBq of ^64^Cu provided only a picomolar concentration of cations, while for ^210^Pb, a 10 nM content of Pb^2+^ was achieved with applied activity. The low chemical content of the cations with sufficient radioactivity, due to the short half-life of medically relevant radionuclides, requires the concentration of ligands for a >95% radiolabeling yield to be determined. Moreover, the presence of additives as pH-adjusting or buffering agents, e.g., acetates, hydrochloric acid, and corresponding impurities, even in highly purified commercially available sources, in the amounts exceeding the radiometal’s content, also impacts the thermodynamic equilibrium. Finally, in highly diluted solutions with low contents of both reactants, i.e., radiometal and ligand, kinetic hindrance for the complexation can also take place.

In the considered systems, a high radiochemical yield of the complexation reaction above 95% was achieved at the H_2_**BA2A1Py** concentration of 50 μM (Table 3) for both [^210^Pb]Pb^2+^ and [^64^Cu]Cu^2+^. Competitive chelation of radiometals by an excess of acetate was initially simulated according to the complexation constants in applied concentrations (Figure S25). It was shown that, even such a significant prevalence of the concentration of acetate can hamper the binding by ligands only at low pH values. It was directly confirmed with regard to the same values of radiolabeling yield obtained for some concentration points with and without NaOAc (Table 3).

It is important to note that in the labeling experiments a solution of the complex was applied on TLC plates immediately after the mixing of the reactants at room temperature. These solutions were additionally analyzed for radiolabeling efficiency after 30 min and 24 h. The same value of radiolabeling yield, irrespective of the duration of synthesis, indicated the rapid kinetics of cation binding (less than a minute).

The formation of radiolabeled complexes, as well as the chemical identity of labeled and stable complexes, was confirmed by TLC and HPLC analysis. The retention time of an LPb stable complex and [^210^Pb]LPb with or without the addition of NaOAc prepared at pH 5–6.5 were the same (t_R_ = 5.2 ± 0.2 min, Figure S26) and proved the identity of the stable complex in a concentration of 2 mM and radiolabeled with c(Pb^2+^) = 10 nM. Additionally, this experiment demonstrated not only the lack of competition of acetate with **BA2A1Py^2−^** for the chelation of radiometals, but also dismissed the possibility of the formation of ternary complexes between radiometal, acetate and ligand that could take place regarding a high content of acetate anion in the radiolabeling process (compared to the ligand and cation). Similarly, in applied TLC conditions, R_f_ of free [^210^Pb]Pb^2+^ or [^64^Cu]Cu^2+^ in the presence of 0.15 M acetate, diverged significantly from ligand-bound species. Additionally, R_f_ and the labeling yield for chelates was the same for radiolabeled complexes prepared with or without 0.15 M AcONa, indicating the formation of the same species irrelative to the presence of acetate.

### 2.8. Stability of Radiolabeled Complexes in Biologically Relevant Media

Taking into account the changes in the stability constants with the cations considered, it is expedient to compare the stabilities of radiolabeled complexes of **BA2A1Py^2−^** with Cu^2+^ and Pb^2+^ in the competing medium, such as serum proteins, because BA3A^3−^ complexes of Cu^2+^ and Pb^2+^ have shown that only the latter was stable enough in this challenging environment, despite the close values of logβ [7,8].

**Figure 7:**
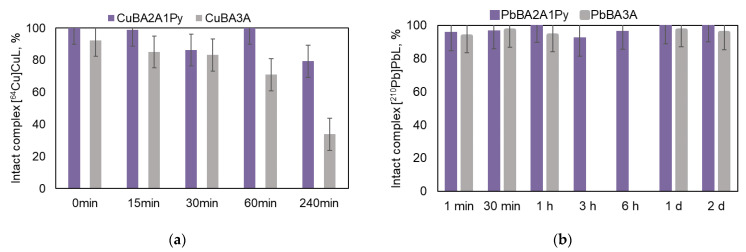
Stability of Cu^2+^ (**a**) and Pb^2+^ (**b**) complexes with BA2A1Py^2−^ compared to complexes with BA3A^3−^ in the presence of serum proteins. Data on M-BA3A complexes are taken from earlier published papers [7,8].

In the nine-fold excess of fetal bovine serum, both complexes of [^64^Cu]Cu**BA2A1Py** and [^210^Pb]Pb**BA2A1Py** have shown a lack of radionuclide loss during 4 h and 2 days, respectively (Figure 7). Comparing the obtained data with earlier published results on complexes with BA3A^3−^ [7,8] it is evident that, despite close complexation constants for CuBA3A and Cu**BA2A1Py**, the stability of the latter radiolabeled chelate is higher. This showed up even after 1 h of incubation and was well pronounced after 4 h. Wherein, both complexes of Pb^2+^ are stable after 2 days of incubation despite more than one order of difference of logβ (Table 1).

Additionally, the stability of complexes was evaluated in the presence of biologically relevant cations such as Fe^3+^, Zn^2+^, Mg^2+^, Ca^2+^ and Cu^2+^ in aqueous solutions and in an isotonic solution of 0.15 M NaCl. The intactness of complexes after incubation for 24 h has been shown by TLC and gamma spectrometry. No loss of radionuclide, in both cases, was detected, meaning the stability of the complexes towards not only transchelation, but also transmetallation.

High stability of both considered complexes with H_2_**BA2A1Py** despite different values of logβ and coordination modes of cations in both complexes should be explained according to individual peculiarities. According to all the obtained results, it is evident that the copper complexes’ stability was mainly governed by the presence of a softer donor atom, while lead complexes’ stability cannot be affected by such substitution even though a complexation constant decreased by a 1.6 order of magnitude. Moreover, shielding of cations from surrounding agents due to pendant arms seems to play an important role for in vitro stability, irrespective of coordination inside or above the macrocycle.

## 3. Discussion

Earlier we have developed a series of azacrown ethers with an aryl substituent in the macrocycle. It was shown that 15-azacrown-5 and 18-azacrown-6 with acetate pendant arms, are appropriate for various medically relevant radiometals [4,5,7,8], but are inappropriate for Cu^2+^. Additionally, the complete substitution of acetate by pyridyl induced a decrease in the water-solubility of the ligands [3]. In this regard, and based on the recent results of DOTAPy complexes with Cu^2+^ [9], in this work we proposed an original and effective method for the synthesis of a hybrid benzoazacrown compound bearing acetate and pyridine pendant arms, providing a water-soluble character and stronger chelation of the copper cation.

Protonation and complexation constants allowed us to fully describe the ligand and Pb^2+^ complex species by NMR spectroscopy. The protonation model is close to the acetate analog H_3_BA3A with macrocyclic symmetric amines, to be the most basic, and the third proton to be associated with the middle pendant arm [8]. Additionally, it is demonstrated that in the acidified solution in the complex, an Pb**HBA2A1Py** cation is held by acetates lacking coordination by the macrocyclic amino groups, similar to the complexation process described for H_4_DOTA [16,17]. Wherein, obviously, due to the larger size of the azacrown cavity providing faster deprotonation of macrocyclic amines, H_2_**BA2A1Py** forms complexes almost immediately, that is confirmed by radiolabeling experiments. These experiments were performed in the pH range where ML complexes should be formed, and according to the calculated structure, supported by EXAFS measurements, Cu^2+^ is coordinated inside the macrocycle **BA2A1Py** capped by pendant arms from both sides, whereas, Pb^2+^ is located above the benzoazacrown ether cavity chelated by all pendant groups from one side, respectively.

Finally, radiolabeled complexes of **BA2A1Py^2−^** and BA3A^3−^ with ^210^Pb turned out to be stable towards transchelation and transmetallation. In the case of ^64^Cu-radiolabeled complexes with BA3A and **BA2A1Py**, only the latter has demonstrated stability in a competing medium, opening perspectives of such a kind of variation of pendant arms for Cu^2+^ chelation. Moreover, regarding the tuning of the water-soluble character of the ligand, the partition coefficient between octanol/water showed a slight decrease in hydrophilicity in H_2_**BA2A1Py**, compared to H_3_BA3A.

## 4. Materials and Methods

All commercially available reagents were used without further purification. The progress of reactions was followed with TLC using aluminum oxide (Merck, Darmstadt, Germany, 60 F254, neutral). Bisamide crown compound **1** [18] and pyridyl derivative **2** [6] were synthesized following the literature procedures.

^1^H and ^13^C NMR spectra were recorded at 25 °C on Bruker Avance 500, Varian Inova 400 MHz spectrometers (Bremen, Germany). Chemical shifts for ^1^H and ^13^C are reported in parts per million (δ) relative to deuterated solvent as an internal reference (CDCl_3_ ^1^H δ = 7.27 and ^13^C δ = 77.00; D_2_O δ = 4.75). Spin–spin coupling constants for the proton spectra (J) are given in hertz (Hz). Spectral assignments were based, in part, on the two-dimensional NMR experiments (COSY, NOESY, HSQC and HMBC). Electrospray ionization mass spectrometry (ESI-MS) analyses were performed using a Finnigan LCQ Advantage (Thermo Fisher Scientific, Waltham, MA, USA) mass spectrometer equipped with an octopole iontrap mass-analyzer, an MS Surveyor pump, a Surveyor auto sampler, a Schmidlin-Lab nitrogen generator (Neuheim, Switzerland) and MestReNova (14.2.0-26256, Santiago de Compostela, Spain, 2020) for data collection and processing. Direct infusion of the sample solution was used. Positive electrospray ionization was achieved using an ionization voltage at 4.5 kV at a temperature of 200 °C. Electrospray full scan spectra in the range *m/z* 100–2000 was obtained by infusion at 0.05 mL·min^–1^ of 50 μM acetonitrile solutions of compounds **3**, and **4** and aqueous solutions of the free ligand H_2_**BA2A1Py**, and in the presence of 2 eq. of Cu(ClO_4_)_2_ and Pb(ClO_4_)_2_. Melting points were determined on a Mel-temp II apparatus in open capillary tubes.

### 4.1. Synthesis and Analysis of Compounds

Compound 3

Bisamide crown compound **2** (309 mg, 0.95 mmol) was dissolved in 1 M BH_3_·THF solution (12 mL) at 0 °C and stirred under an inert atmosphere overnight. Excess BH_3_∙THF was destroyed by adding of MeOH (10 mL). The solvent was evaporated under vacuum. Then, 1 M HCl (10 mL) was added to the residue and the reaction mixture was refluxed for 8 h. The solution was washed with CH_2_Cl_2_, and then the pH was adjusted to 9–10 by adding NaOH. The product was extracted with CH_2_Cl_2_. The solvent was evaporated under vacuum to give a brown oil (206 mg, yield 61%). ^1^H NMR (CDCl_3_, 400 MHz, 25 °C): 2.73 (t, 4H, H(7), *J* = 4.3, 5.1), 2.84–2.89 (m, 8H, H(5,6)), 3.79 (s, 2H, H(8)), 4.10 (t, 4H, H(4), *J* = 4.3, 4.7), 6.97 (s, 4H, H(1,2)), 7.08 (t, 1H, H(12), *J* = 5.9, 6.3), 7.48 (t, 1H, H(11), *J* = 7.0, 7.8), 7.52 (d, 1H, H(10), *J* = 7.4), 8.45 (d, 1H, H(13), *J* = 4.3). ^13^C NMR (CDCl_3_, 400 MHz, 25 °C): 47.16 (C-7), 48.82 (C-5), 54.97 (C-6), 60.60 (C-8), 69.22 (C-4), 115. 39 (C-2), 122.02 (C-1,12), 122.92 (C-10), 136.96 (C-11), 148.94 (C-13), 149.34 (C-3), 160.12 (C-9). MS (ESI): *m/z* [M + H]^+^ calcd for C_20_H_28_N_4_O_2_ + H^+^: 357.43; found: 357.3. Anal. Calcd for C_20_H_28_N_4_O_2_·3H_2_O: C, 58.52; H, 8.35; N, 13.65%. Found: C, 58.40; H, 8.51; N, 13.58%.

Compound 4

A solution of *tert*-butyl bromoacetate (363 mg, 1.86 mmol) in MeCN (5 mL) was added to a mixture of **3** (332 mg, 0.93 mmol), K_2_CO_3_ (513 mg, 3.72 mmol) and KI (308 mg, 1.86 mmol) in MeCN (15 mL) and refluxed for 15 h. The solvent was evaporated under vacuum and the residue was dissolved in H_2_O and extracted with CHCl_3_. The crude product obtained by concentration of the combined organic extracts was purified by column chromatography (Al_2_O_3_, EtOAc/MeOH 10:1). The product was obtained as a brown oil (250 mg, yield 46%). ^1^H NMR (CDCl_3_, 400 MHz, 25 °C): 1.41 (s, 18H, H(11)), 2.77 (t, 4H, H(7), *J* = 5.9, 7.0), 3.02 (t, 4H, H(6), *J* = 6.6, 7.4), 3.13 (t, 4H, H(5), *J* = 3.9), 3.27 (s, 4H, H(8)), 3.79 (s, 2H, H(12)), 4.06 (t, 4H, H(4), *J* = 3.9), 6.82–6.88 (m, 4H, H(1, 2)), 7.12 (t, 1H, H(16), *J* = 5.9, 6.3), 7.46 (d, 1H, H(14), *J* = 7.8), 7.60 (t, 1H, H(15), *J* = 7.4), 8.48 (d, 1H, H(17), *J* = 5.1). ^13^C NMR (CDCl_3_, 400 MHz, 25 °C): 28.10 (C-11), 52.91 (C-7), 52.94 (C-6), 53.43 (C-5), 56.93 (C-8), 61.69 (C-12), 68.33 (C-4), 80.77 (C-10), 112.20 (C-2), 120.63 (C-1), 121.73 (C-16), 122.82 (C-14), 136.28 (C-15), 148.56 (C-3), 148.80 (C-17), 160.16 (C-13), 170.91 (C-9). MS (ESI): *m/z* [M + H]^+^ calcd for C_32_H_48_N_4_O_6_ + H^+^: 585.37; found: 585.5; *m/z* [M + Na]^+^ calcd for C_32_H_48_N_4_O_6_ + Na^+^: 607.35; found: 607.48. Anal. Calcd for C_32_H_48_N_4_O_6_·2H_2_O: C, 61.91; H, 8.44; N, 9.03%. Found: C, 61.85; H, 8.53; N, 9.08%.

Compound H_2_**BA2A1Py**

Ditert-butyl ester 4 (250 mg, 0.43 mmol) was dissolved in H_2_O (10 mL) and refluxed for 18 h. The solvent was evaporated under vacuum. The product was obtained as a dark yellow solid (199 mg, yield 98%). Mp.165–168 °C. ^1^H NMR (D_2_O, 500 MHz, 25 °C): 2.99 (br.s, 4H, H(7)), 3.61 (br.s, 4H, H(6)), 3.64 (br.s, 4H, H(5)), 3.68 (br.s, 2H, H(10)), 3.77 (s, 4H, H(8)), 4.31 (br.s, 4H, H(4)), 6.99 (br.s, 4H, H(1,2)), 7.29 (t, 1H, H(14), *J* = 5.3, 7.2), 7.41 (d, 1H, H(12), *J* = 6.0), 7.75 (t, 1H, H(13), *J* = 7.3), 8.35 (d, 1H, H(15), *J* = 4.6). ^13^C NMR (D_2_O, 500 MHz, 25 °C): 49.10 (C-7, C-10), 53.37 (C-5, C-6), 55.90 (C-8), 61.50 (C-4), 112.93 (C-2), 122.27 (C-1), 123.69 (C-14), 124.94 (C-12), 139.07 (C-13), 146.52 (C-3), 148.07 (C-15), 154.72 (C-11), 169.69 (C-9). MS (ESI): *m/z* [M + H]+ calcd for C_24_H_32_N_4_O_6_ + H+: 473.24; found: 473.3; *m/z* [M + Na]+ calcd for C_24_H_32_N_4_O_6_ + Na+: 495.22; found: 495.19.; *m/z* [M + K]+ calcd for C_24_H_32_N_4_O_6_ + K+: 511.2; found: 511.2. Anal. Calcd for C_24_H_32_N_4_O_6_·2,5H_2_O: C, 55.69; H, 7.21; N, 10.83%. Found: C, 55.68; H, 7.12; N, 10.55%.

### 4.2. Potentiometric Titration

Potentiometric titration was performed using an 848 Titrino Plus autotitrator (Metrohm, Herisau, Switzerland) equipped with a 5 mL autoburette and Metrohm combined pH glass electrode (model 60262100). A constant temperature of 25.0 ± 0.1 °C in the titration vessel was maintained with a thermostat throughout the experiments. Nitrogen flow through the titration vessel was used in all experiments. A combined glass electrode was calibrated by titration of a preliminarily standardized solution of HClO_4_ by NaOH solution with a known concentration. The calculation of the standard electrode potential, E_0_, and the slope of electrode function, was performed using the GLEE program (glass electrode evaluation) according to the Gran method [19]. The ionic product of water, pKw = 13.77, was taken for 0.1 M KNO_3_. For determination of the ligand’s protonation constants, potentiometric titration of a 0.001 M H_2_**BA2A1Py** solution in the presence of 0.01 M HClO_4_ was carried and the protonation constants were calculated with the HyperQuad2008 program. To determine the stability constants of Pb^2+^ and Cu^2+^ complexes with H_2_**BA2A1Py**, titration of a 0.001 M ligand solution was carried out in the presence of an equimolar amount of a Pb^2+^ or Cu^2+^, or two-fold excess of Cu^2+^, and a 0.01 M HClO_4_. To maintain constant ionic strength during an experiment a solution of 0.1 M KNO_3_/NaClO_4_ was used as a background electrolyte. The stability constants of complexes were calculated with the HyperQuad2008 program using the hydrolysis constants, the ionic product of water, the preliminarily determined ligand’s protonation constants and E_o_ value [15].

### 4.3. NMR Study of Ligand H_2_BA2A1Py Protonation and Complex Formation with Pb^2+^ Ion

Ligand **H_2_BA2A1Py** samples were prepared in D_2_O at various pD values for NMR studies of protonation. The pD values were selected in accordance with the data of potentiometric titration to obtain spectra of various protonated forms (L^2−^, HL^−^, H_2_L, H_3_L^+^) by adding small volumes of concentrated solutions of NaOH and HClO_4_. Samples of the Pb^2+^ complex for the NMR measurements was prepared by dissolving 1 eq. ligand and 2 eq. Pb(ClO_4_)_2_ in D_2_O, followed by adjustment of the desired pD with small volumes of concentrated NaOH and HClO_4_. The accurate pD measurements in D_2_O were obtained by a direct reading in a D_2_O solution using a combined glass/AgCl electrode after appropriate calibration procedures by usage of standard buffers.

### 4.4. Determination of logP

An aqueous solution of the corresponding ligand 0.5 mM, 0.05 M NaOAc and 4 kBq of ^210^Pb at pH5.5 were mixed with an equal volume of n-octanol (Sigma, Roedermark, Germany) (500 μL/500 μL) and resulting samples were thoroughly shaken for 20 min at room temperature. After centrifugation, organic and aqueous phases were separated and measured on a gamma-spectrometer. The *logP* was calculated under the following conditions of the corresponding ligand, 0.05 M NaOAc and 4 kBq of ^210^Pb at pH5.5 were mixed with an equal volume of n-octanol (Sigma, Roedermark, Germany) (500 μL/500 μL) and resulting of samples were thoroughly shaken for 20 min at room temperature. After centrifugation, organic and aqueous phases were separated and measured on gamma spectrometer. The *logP* was calculated as follows

*logP* = *A(oct)*/*A(aq),*

where *A(oct)*—count rate of ^210^Pb in the octanol’s phase, *A(aq)*—count rate of ^210^Pb in the aqueous phase.

### 4.5. High Performance Liquid Chromatography (HPLC)

Analytical HPLC was performed on a Waters system (Milford, MA, USA) equipped with a Waters 1525 binary HPLC pump, Waters C-18 reversed-phase column (4.6 × 75 mm), Waters 2489 UV-Vis detector and Waters Fraction collector III, using following modes:

For the copper complex: isocratic total flow 1 mL/min, A—0.01%TFA in mQ water—0.85 mL/min, B—CH_3_OH—0.15 mL/min

For the lead complex: isocratic total flow 1 mL/min, A—mQ water—0.9 mL/min, B—CH_3_CN—0.1 mL/min

Retention times of non-radioactive complexes Pb**BA2A1Py** (2 mM) and Cu**BA2A1Py** (1.7 mM) prepared at pH 5–7 were 5.2 ± 0.4 min and 5.3 ± 0.2 min, respectively. Detection of stable complexes was performed via a UV/Vis absorption at 267 nm (Figure S26). Retention time of radiolabeled [^210^Pb]Pb**BA2A1Py** complex was the same: activity peaked on fractions of 5 and 5.5 mL, as it was shown by measuring the activity of fractions collected every 0.5 min, using a gamma spectrometer (Figure S26).

### 4.6. DFT Calculations

The calculations were performed at the DFT level of theory [20,21] with PBE0 hybrid functional [22,23] and dispersion correction D4 [24]. The atomic orbitals were modelled with the def2-tzvp basis set [25] (and the corresponding effective core potential for the Pb^2+^ ion [26]). All the calculations were performed in the Orca5 package [27].

### 4.7. Radiochemical Separation of ^210^Pb

Radiochemical separation of ^210^Pb using Sr-resin (Triskem Int. France) is described in the literature [28]. Briefly, a solution of ^226^Ra dissolved in 0.1 M HNO_3_ was loaded on a Sr-resin chromatographic column, a solution of 0.1 M HNO_3_ was passed through a column until the radium was completely rinsed, then ^210^Pb was washed off with 0.05 M (NH_4_)_3_cit. The separation of ^210^Pb from ^226^Ra was monitored by using gamma spectrometry (with an HPGe-detector GR3818 Canberra Ind.) at 46.5 keV (^210^Pb) and 186.2 keV (^226^Ra) peaks. The resulting solution was evaporated and redissolved in 0.1 M HCl.

### 4.8. Production and Radiochemical Separation of ^64^Cu

Nickel target was irradiated using the cyclotron at the Skobeltsyn Institute of Nuclear Physics, Moscow State University. The ^64^Cu was produced according to the ^64^Ni(p,n)^64^Cu reaction [29] from a nickel metal target (0.5 g) of natural isotopic composition using an 8-MeV proton beam at 1 μA for 3.5 h. Separation was performed on Cu Resin (Triskem), as previously described [30]. The resulting carrier-free radionuclide solutions in each experiment was added along with the solution of stable Cu(ClO_4_)_2_.

### 4.9. Radiolabeling Studies

Radionuclide stock solutions for labeling were prepared in 0.1 M HCl (PanReac AppliChem, Darmstadt, Germany, HPLC grade diluted in deionised water): (a) ^210^Pb stock solution 45 kBq/mL; (b) ^64^Cu stock solution 10 kBq/mL, samples 2.5 kBq/mL or 2.7 × 10^−13^ M.

The complex was labeled at room temperature in the 0.15 M NaOAc at pH5–6. For radiolabeling experiments, 4 kBq of ^210^Pb and 1 kBq of ^64^Cu per sample were used, leading to a final concentration of cation to be 20 nM and 0.3 pM, respectively. In the case of the copper complex, a stable Cu^2+^ carrier was added to reach the concentration 10 nM of cations. Thin layer chromatography (TLC) was used to control the bound fraction of the radionuclide. TLC experiments were performed using cellulose on aluminum plates (Sigma) as a stationary phase and 10 mM NaOH dissolved in 0.9% NaCl as an eluent for the ^210^Pb-labeled complex and silica gel on aluminum plates (Sigma) and 10% AcONH_4_:CH_3_OH (1:1) for ^64^Cu-labeled complex. Analysis of the distribution of radioactivity on the plates was carried out using gamma spectroscopy.

To select the optimal concentration of ligand, the ^210^Pb solution was mixed with 0.15 M NaOAc and various concentrations of ligand; the degree of complex formation was determined by the TLC method described above, followed by gamma spectrometry of the TLC plates. To study the labeling as a function of pH, solutions of the complex were prepared in the same way with the addition of 0.5 M HCl for pH 2.5 and 0.1 M NaOH for pH 10.5.

### 4.10. Stability of Complexes in Biologically Relevant Media

The resulting complex with a radiochemical purity of >95% was added to solutions of microelements (5 mM Ca^2+^ and Mg^2+^, 0.1 mM Fe^3+^, Zn^2+^ and Cu^2+^) and isotonic solution of 0.15 M NaCl. The bound radionuclide fraction was measured by TLC and gamma spectroscopy after 24 h of incubation at 37 °C.

For the serum stability experiment, labeled compounds with a higher activity content, 40 kBq ^210^Pb and 10 kBq ^64^Cu, were synthesized. The obtained complexes were mixed with a nine-fold excess of fetal bovine serum (HyClone, Logan, UT, USA) and incubated at 37 °C, then an aliquot of this solution was taken at fixed time points and the protein fraction was precipitated with an excess of ethanol, cooled to 2–4 °C, centrifuged and the supernatant was separated. The content of radioactivity in the supernatant was established using gamma spectrometry. The experiment, without an addition of ligand, was carried out in the same conditions.

### 4.11. EXAFS Measurement and Data Treatment

The Cu K- and Pb L_3_- edges X-ray spectra for samples of corresponding aqueous solutions containing 0.005 M of complex, at pH 2.3 and 7.6 for Cu^2+^ and pH2.4 and 5.1 for Pb^2+^, were collected at the beamline “Structural Materials Science” [31] using the equipment of “Kurchatov Synchrotron Radiation Source” (Moscow, Russia). The storage ring with electron beam energy of 2.5 GeV and a current of 80–100 mA was used as the source of radiation. All the spectra were collected in the transmission mode using a Si (111) channelcut monochromator. EXAFS data (*χ_exp_(k)*) were analyzed using the IFEFFIT data analysis package [32]. EXAFS data reduction used standard procedures for the pre-edge subtraction and spline background removal. The Fourier transformation (FT) of the k^2^ weighted EXAFS functions *χ_exp_(k)* was calculated over the range of photoelectron wave numbers *k* = 2–11.0 Å^−1^ (for both complexes). The structural parameters, including interatomic distances (R_i_), coordination numbers (N_i_) and Debye–Waller factors (σ^2^), were found by the non-linear fit of theoretical spectra (eq.) to experimental ones.

$$\chi \left(k\right){=S}_{0}^{2}\sum_{i=1}^{n}\frac{N_{i}F_{i}\left(k\right)}{R_{i}^{2}k}e^{\frac{{-2R}_{i}}{\lambda \left(k\right)}}e^{{-2\sigma }_{i}^{2}k^{2}}\sin\left({2\mathrm{kR}}_{i}{+F}_{i}\left(k\right)\right)$$

The theoretical data were simulated using the photoelectron mean free path *λ(k)*, amplitude *F_i_(k)*, and phase shift *F_i_(k)* calculated ab-initio using the program FEFF6 [33].

## Figures and Tables

**Table 1:** Protonation constants of BA2A1Py^2−^ and the closest analog BA3A^3−^ and stability constants of the studied complexes.

Cation	Complex *^a^*	BA2A1Py^2−^		BA3A^3−^ [7,8]
		0.1 M NaClO_4_	0.1 M KNO_3_	0.1 M KNO_3_
H^+^	HL	9.0 (1)	9.3 (1)/9.3	9.9 (1)/9.9
	H_2_L	16.7 (1)	17.0 (1)/7.7	17.7 (1)/7.8
	H_3_L	21.2 (1)	20.8 (1)/3.8	21.2 (1)/3.5
	H_4_L	22.9 (1)	22.5 (1)/1.7	23.8 (2)/2.6
Cu^2+^	HLCu	21.2 (1)	20.0 (1)	20.4 (1)
	LCu	16.2 (2)	16.3 (1)	16.8 (1)
	LCuOH	18.8 (2)	18.6 (1)	
Pb^2+^	HLPb	-	18.6 (1)	20.1 ± 0.1
	LPb	-	15.6 (1)	17.2 ± 0.1
	LPbOH	-	20.2 (2)	23.1 ± 0.1
	LPb(OH)_2_	-	22.4 (2)	26.3 ± 0.1

*^a^* charges are omitted for clarity.

**Table 2:** Structural parameters refined from fitting the EXAFS Cu K- and Pb L3- edges spectra for Cu**BA2A1Py** and Pb**BA2A1Py** complexes solutions at varied pHs.

Sample	N/O			C/N/O			C/N/O			C/N/O		
	N	R, Å	σ, Å	N	R, Å	σ, Å	N	R, Å	σ, Å	N	R, Å	σ, Å
Cu**BA2A1Py**_pH2.3	4.0	2.00	0.011	4.6	2.72	0.005	6.9	2.94	0.005	1.6	3.29	0.005
Cu**BA2A1Py**_pH7.6	4.0	1.99	0.010	3.4	2.73	0.005	5.7	2.92	0.005	2.2	3.41	0.005
Pb**BA2A1Py**_pH2.4	5.5	2.54	0.014	6.6	3.31	0.009	-					
Pb**BA2A1Py**_pH5.1	6.1	2.55	0.016	8.6	3.31	0.009						

**Table 3:** Labeling efficiency (%) of complexes at different ligand concentrations.

c(H_2_BA2A1Py), μM	PbL, %, 0.15 M Ac (No Ac)	CuL, %, 0.15 M Ac
1000	100 ± 7	97 ± 9
500	100 ± 9	92 ± 8
200	99 ± 9	–
100	100 ± 8 (99%)	99 ± 2
80	99 ± 7	–
50	95 ± 9 (99%)	96 ± 3
20	73 ± 7	–
10	62 ± 3	34 ± 2

## Supplementary Materials

The following supporting information can be downloaded at: https://www.mdpi.com/article/10.3390/molecules27103115/s1, Figure S1. ^1^H and ^13^C NMR spectra of 3 in CDCl_3_; Figure S2. COSY spectrum of 3 in CDCl_3_; Figure S3. NOESY spectrum of 3 in CDCl_3_; Figure S4. HSQC spectrum of 3 in CDCl_3_; Figure S5. HMBC spectrum of 3 in CDCl_3_; Figure S6. MS (ESI) spectrum of 3; Figure S7. ^1^H and ^13^C NMR spectra of 4 in CDCl_3_; Figure S8. COSY spectrum of 4 in CDCl_3_; Figure S9. NOESY spectrum of 4 in CDCl_3_; Figure S10. HSQC spectrum of 4 in CDCl_3_; Figure S11. HMBC spectrum of 4 in CDCl_3_; Figure S12. MS (ESI) spectrum of 4; Figure S13. ^1^H and ^13^C NMR spectra of H_2_BA2A1Py in D_2_O (pD = 5.7); Figure S14. COSY spectrum of H_2_BA2A1Py in D_2_O (pD = 5.7); Figure S15. NOESY spectrum of H_2_BA2A1Py in D_2_O (pD = 5.7); Figure S16. HSQC spectrum of H_2_BA2A1Py in D_2_O (pD = 5.7); Figure S17. HMBC spectrum of H_2_BA2A1Py in D_2_O (pD = 5.7); Figure S18. MS (ESI) spectrum of H_2_BA2A1Py; Figure S19. COSY spectrum of BA2A1Py^2−^ in D_2_O (pD = 11.2); Figure S20. NOESY spectrum of BA2A1Py^2−^ in D_2_O (pD = 11.2); Figure S21. ^13^C NMR spectrum of H_2_BA2A1Py in the presence of Pb^2+^ (*C*(L) = 10 мM, pD = 6.3) in D_2_O; Table S1. ^1^H NMR chemical shifts (Δδ, ppm) of H_2_BA2A1Py recorded in D_2_O solution in the absence and presence of Pb^2+^; Figure S22. MS (ESI) spectrum of H_2_BA2A1Py in the presence of Pb^2+^ in water; Figure S23. MS (ESI) spectrum of of H_2_BA2A1Py in the presence of Cu^2+^ in water; Figure S24. Potentiometric titration curves of solutions, containing 1 mM of L and 1 mM of L with 1 mM of M^2+^ in 0.1 M KNO_3_ (a) 0.1M NaClO_4_ (b) and 1 mM of L with 2 mM of Cu^2+^ in 0.1 M KNO_3_; Figure S25. Species distribution diagrams in the systems: (a) Pb^2+^ (10 nM), H_2_BA2A1Py (0.1 mM), Ac^−^ (0.15 M); (b) Cu^2+^ (10 nM), H_2_BA2A1Py (0.1 mM), Ac^−^ (0.15 M); Figure S26. Chromatograms of: (a) Cu-BA2A1Py (non-radioactive, 1.7 mM, pH5.3) recorded by UV-Vis detector at 267 nm; (b) Pb-BA2A1Py (non-radioactive, 2 mM, pH7.4) recorded by UV-Vis detector at 267 nm; (c) [^210^Pb]Pb-BA2A1Py (radioactive, c(L) = 0.1 mM, pH5.3, without NaOAc) plotted according to measured activity in the collected fractions (correction to dead volume was applied); Figure S27. Chromatogram of AcONa (0.15 M) recorded by UV-Vis detector at 221 nm (a) and 267 nm (b), in the mode used for Pb-BA2A1Py complex (isocratic, H_2_O – 0.9, CH_3_CN – 0.1).

## Sample Availability

Samples of the compounds are available from the authors.
