# Supplementary material for: Novel Hybrid Benzoazacrown Ligand as a Chelator for Copper and Lead Cations: What Difference Does Pyridine Make
Source: Molecules. 2022 May 12;27(10):3115. doi: 10.3390/molecules27103115 (PMC9143346; doi:10.3390/molecules27103115)
Supplement: Supplementary file 1 [file molecules-27-03115-s001.zip › molecules-1691388-supplementary.pdf]

Electronic Supplementary Information for

Novel hybrid benzoazacrown ligand as chelator for Lead and Copper cations: what the difference a pyridine makes

by B.V. Egorova, L.S. Zamurueva, A.D. Zubenko, A.V. Pashanova, A.A. Mitrofanov, A.B. Priselkova, Yu.V. Fedorov, A.L. Trigub, O.A. Fedorova and S.N. Kalmykov

Content

NMR and MS characterization of ligand, intermediate products and considered complexes 1

Potentiometric titration curves 14

pH distribution diagrams of cations' species in presence of ligand and acetate 14

HPLC chromatograms 15

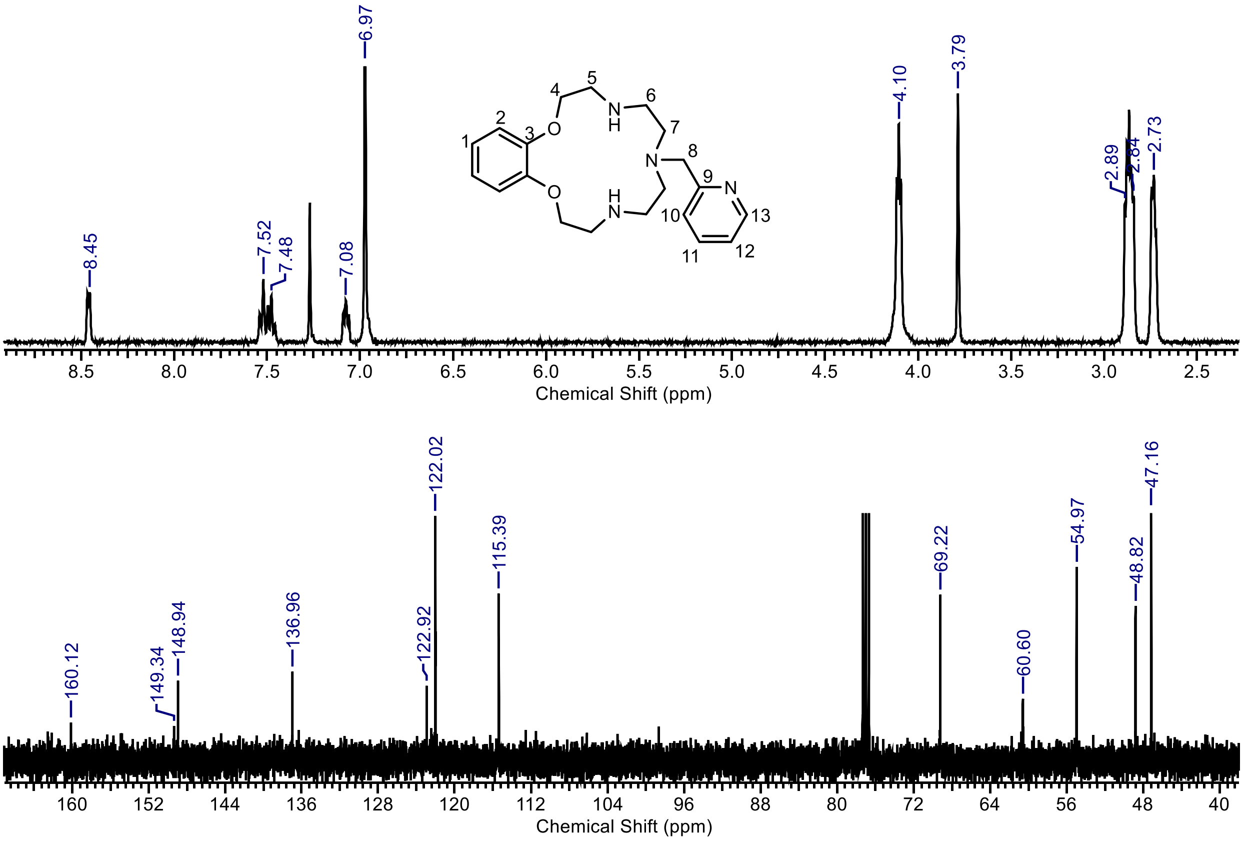

Figure S1. ^{1}H and ^{13}C NMR spectra of **3** in $CDCl_3$.

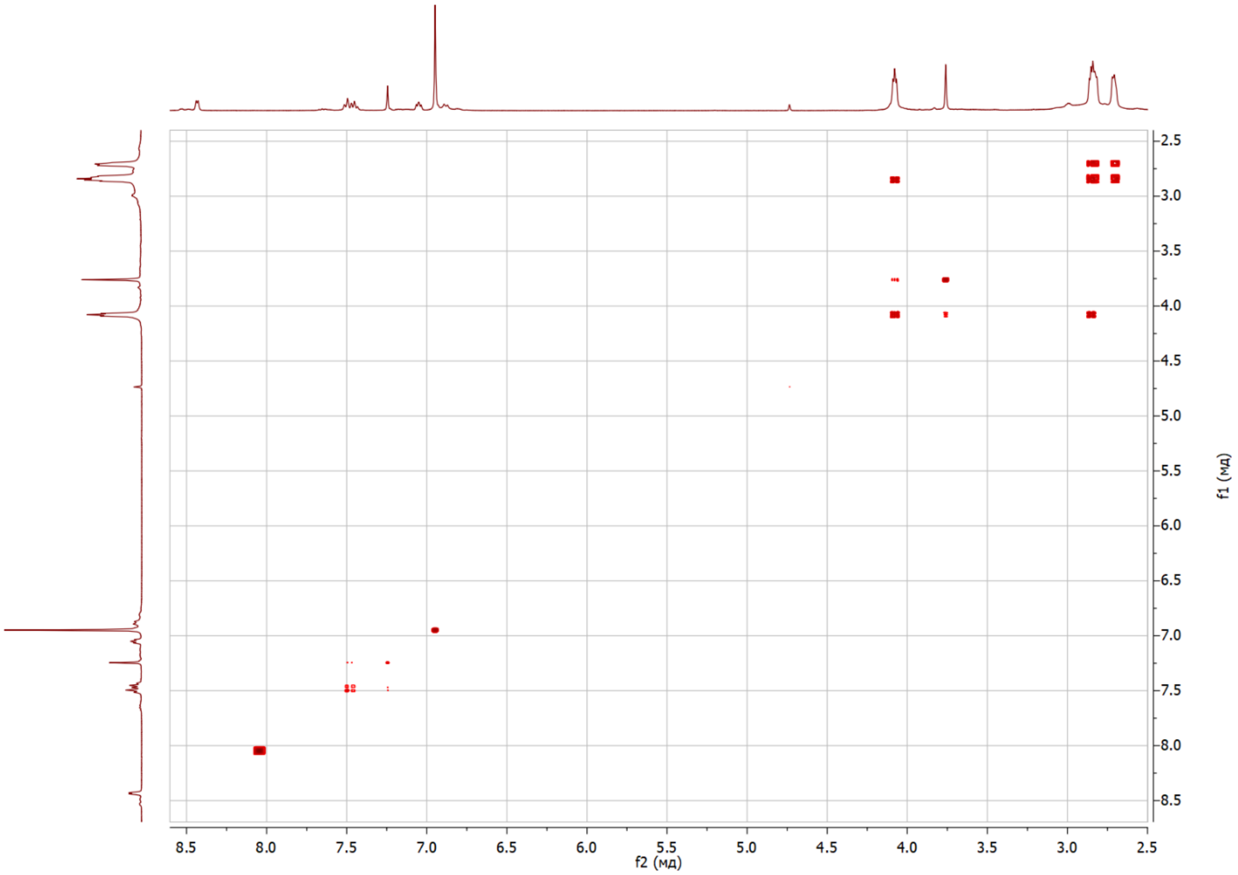

Figure S2. COSY spectrum of **3** in $CDCl_3$.

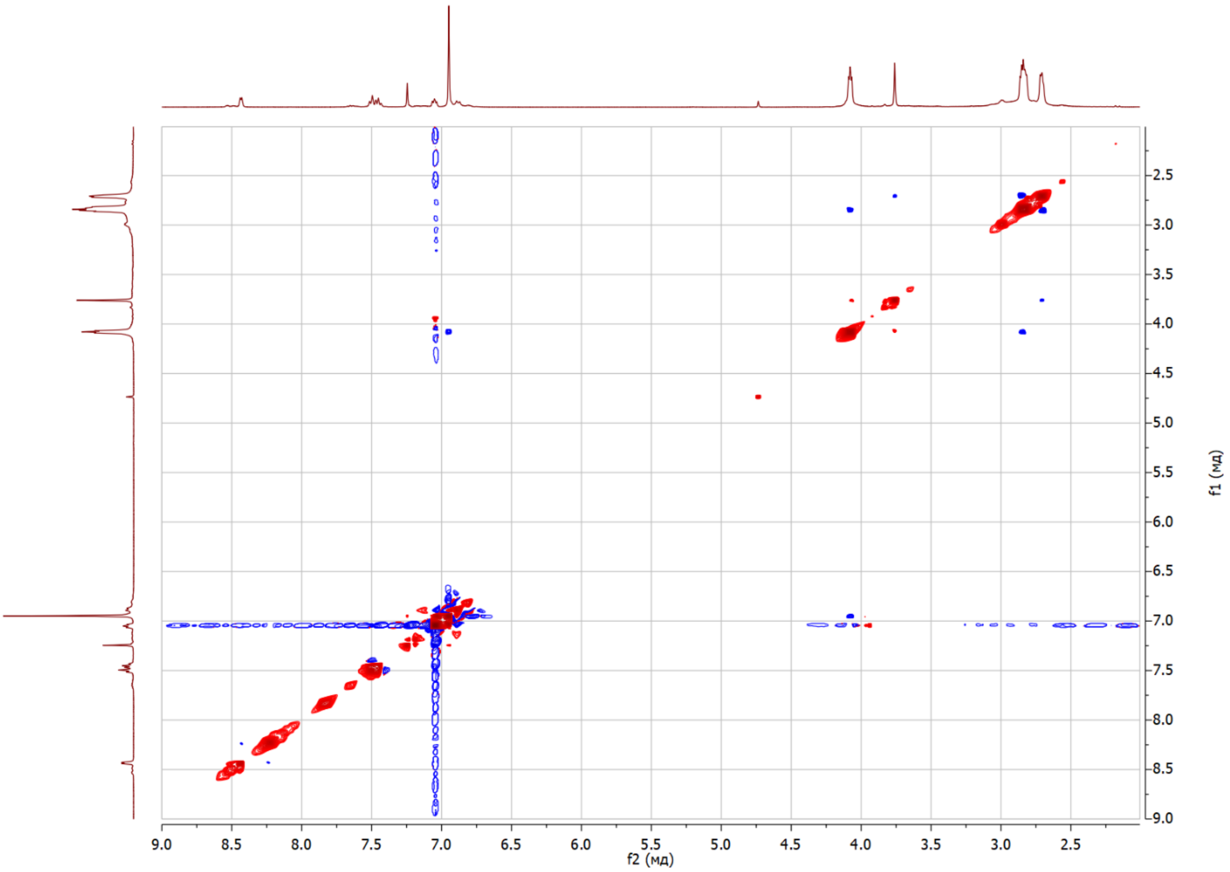

Figure S3. NOESY spectrum of **3** in $CDCl_3$.

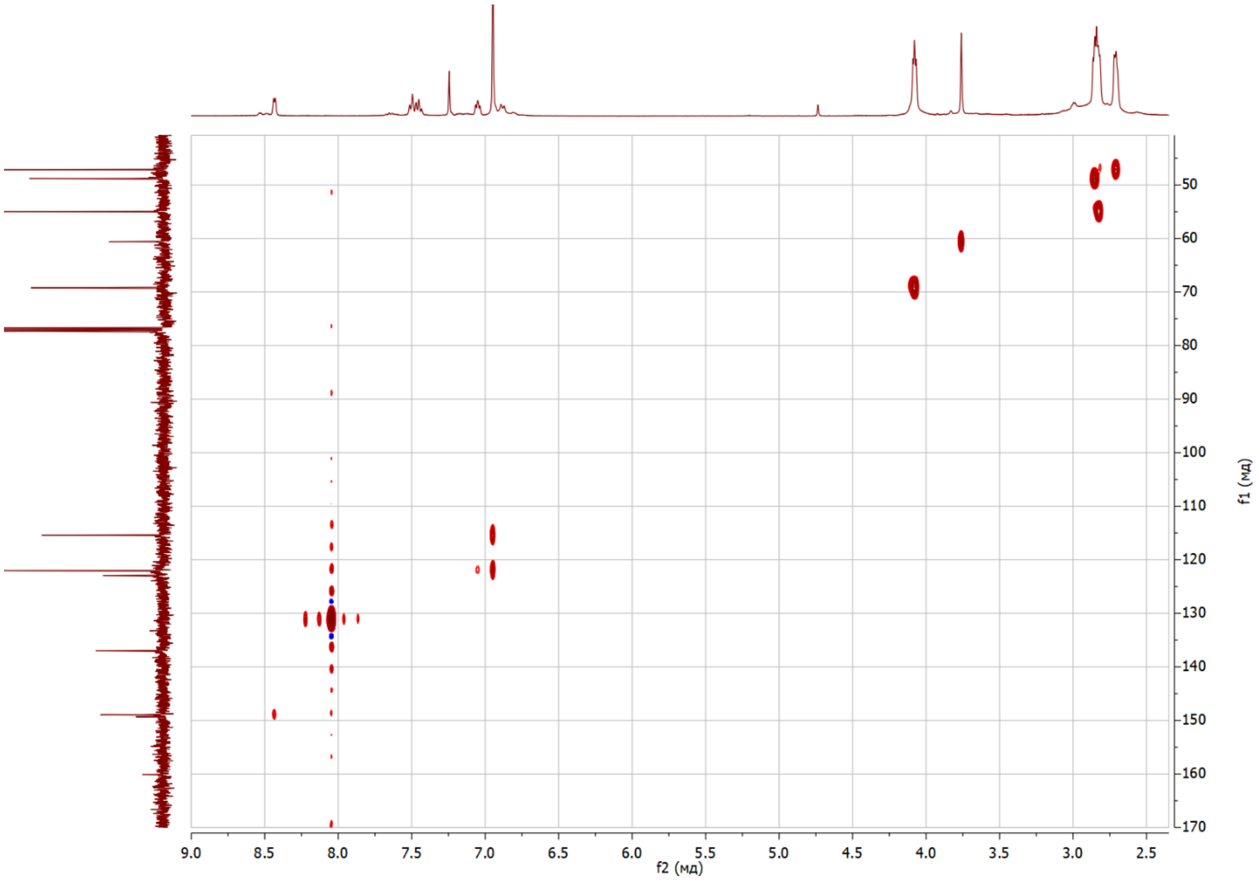

Figure S4. HSQC spectrum of **3** in $CDCl_3$.

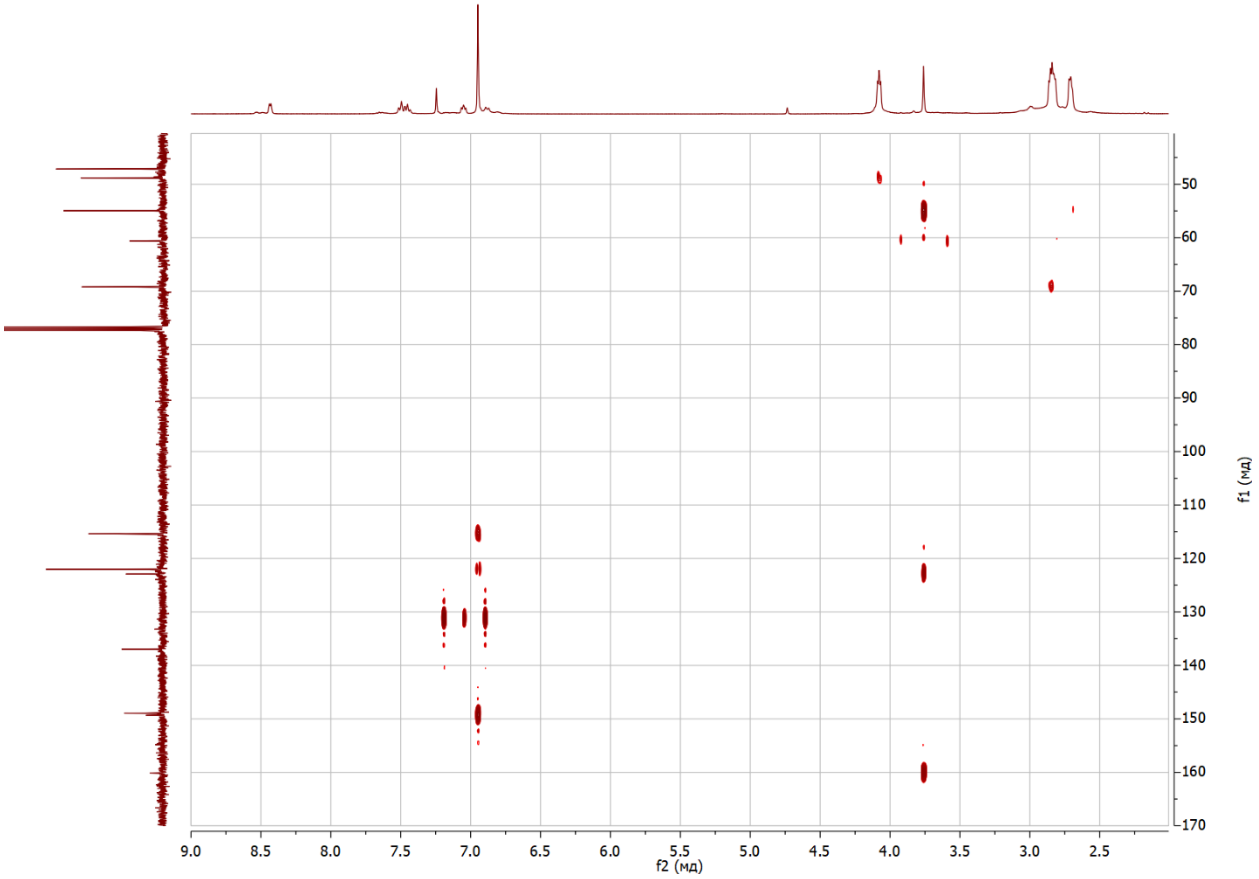

Figure S5. HMBC spectrum of **3** in $CDCl_3$.

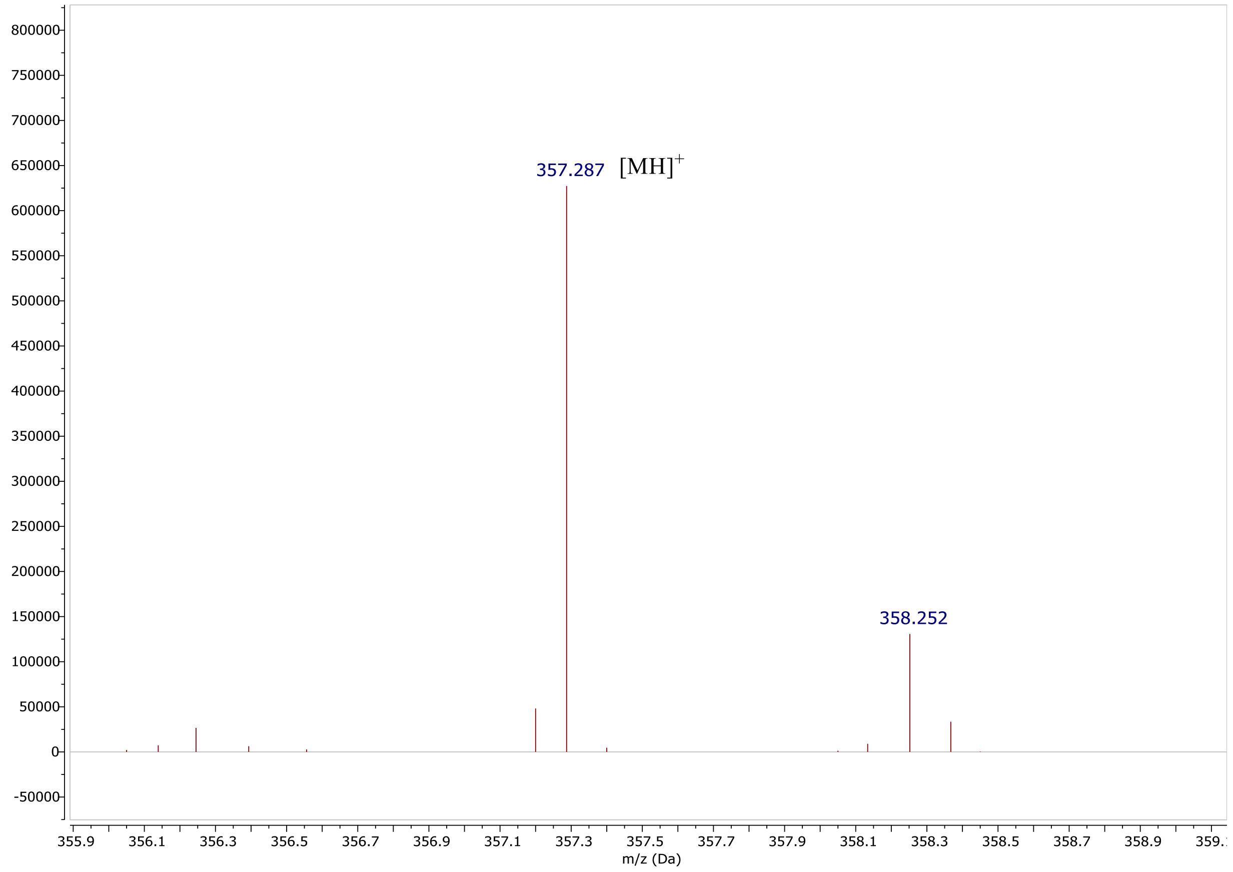

Figure S6. MS (ESI) spectrum of **3**.

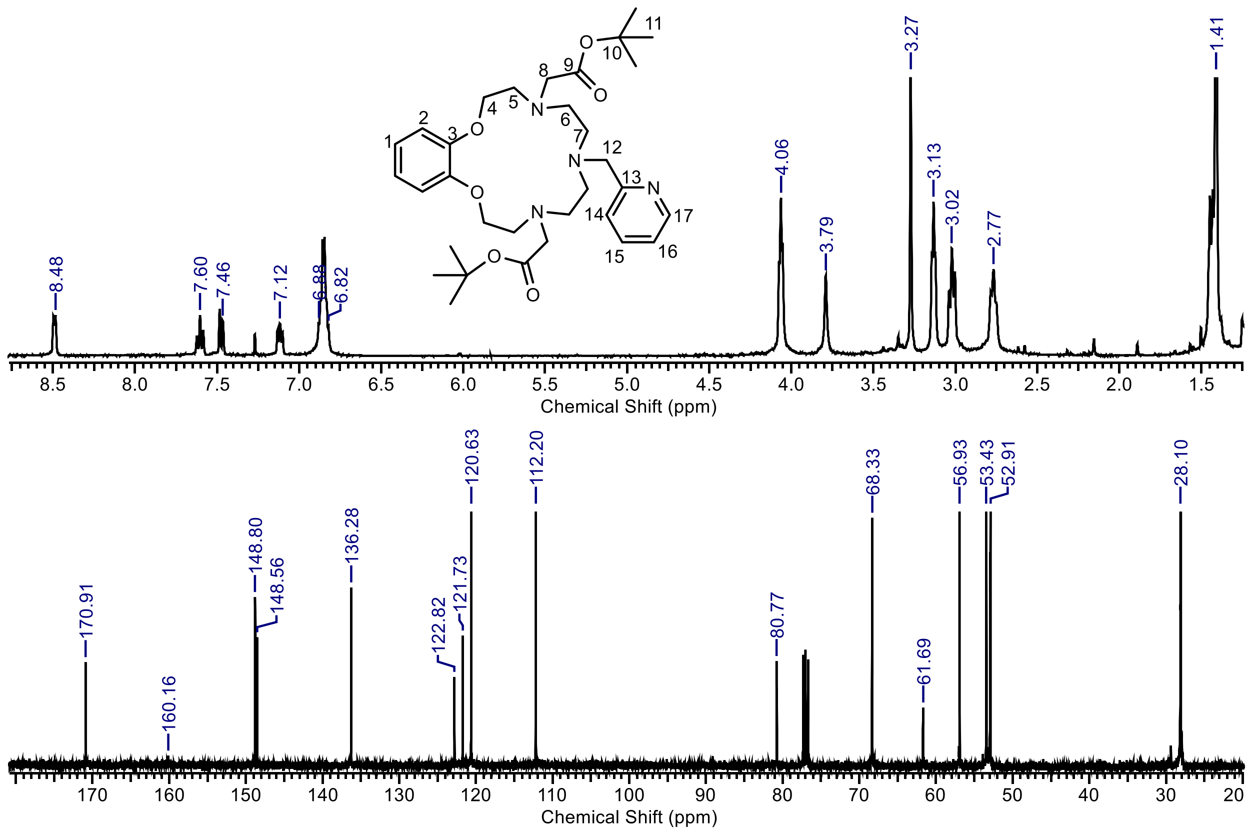

Figure S7. ^{1}H and ^{13}C NMR spectra of **4** in $CDCl_3$.

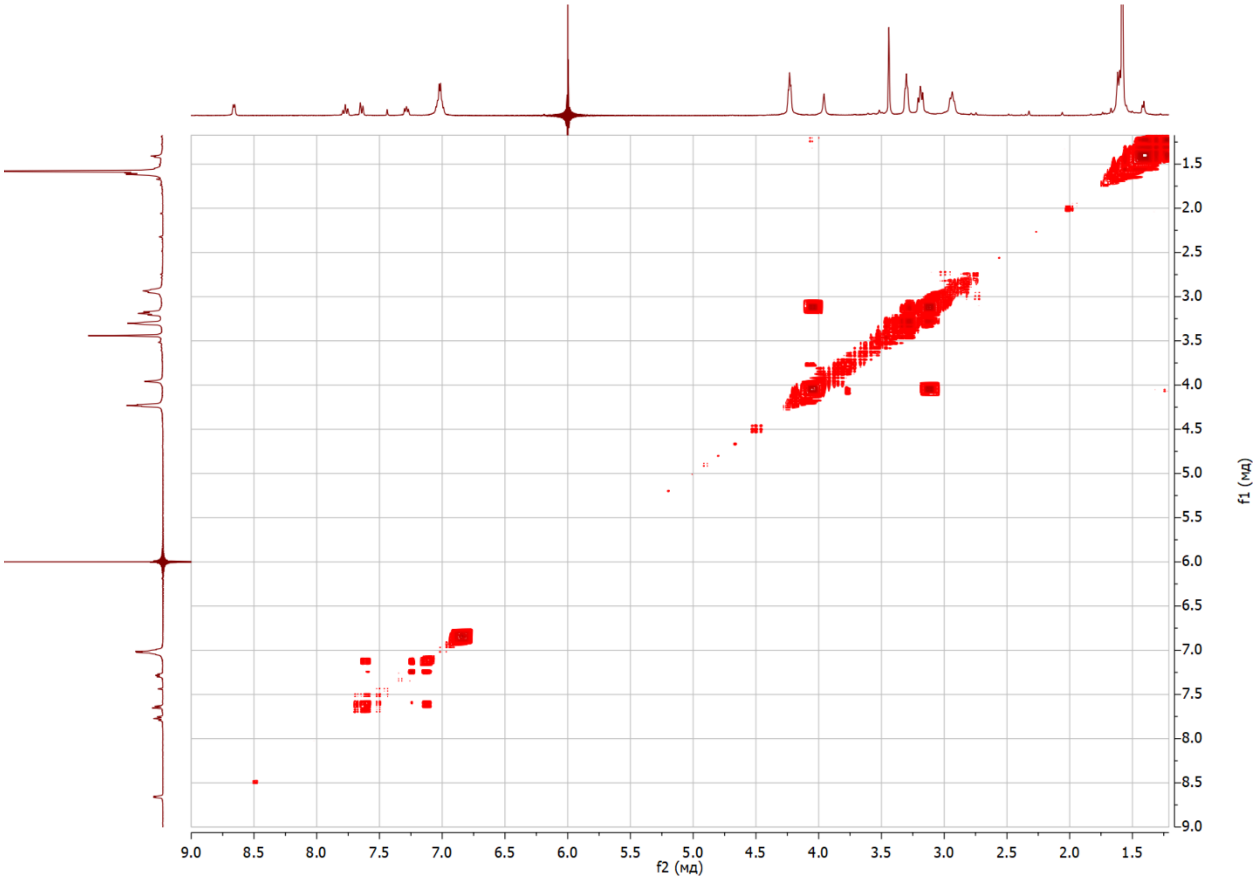

Figure S8. COSY spectrum of **4** in $CDCl_3$.

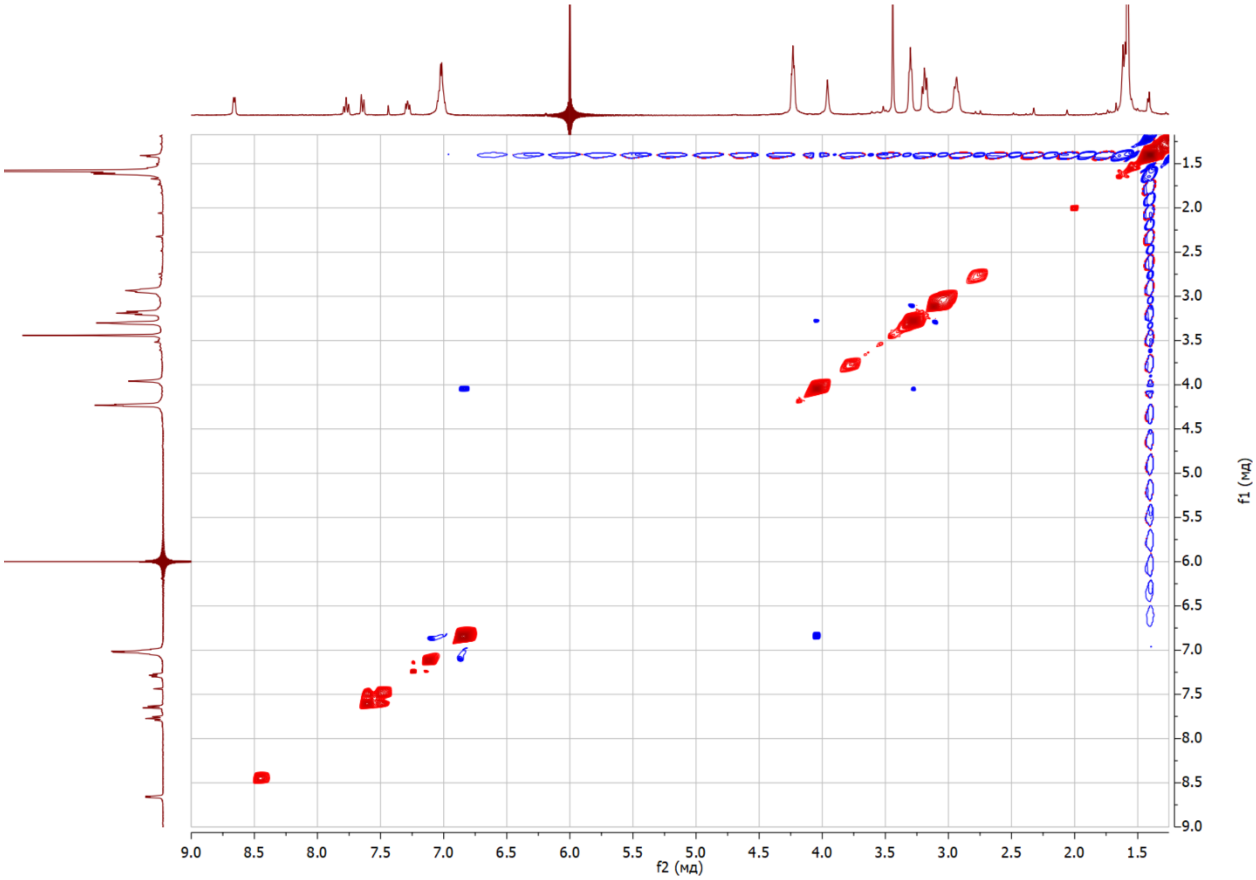

Figure S9. NOESY spectrum of **4** in $CDCl_3$.

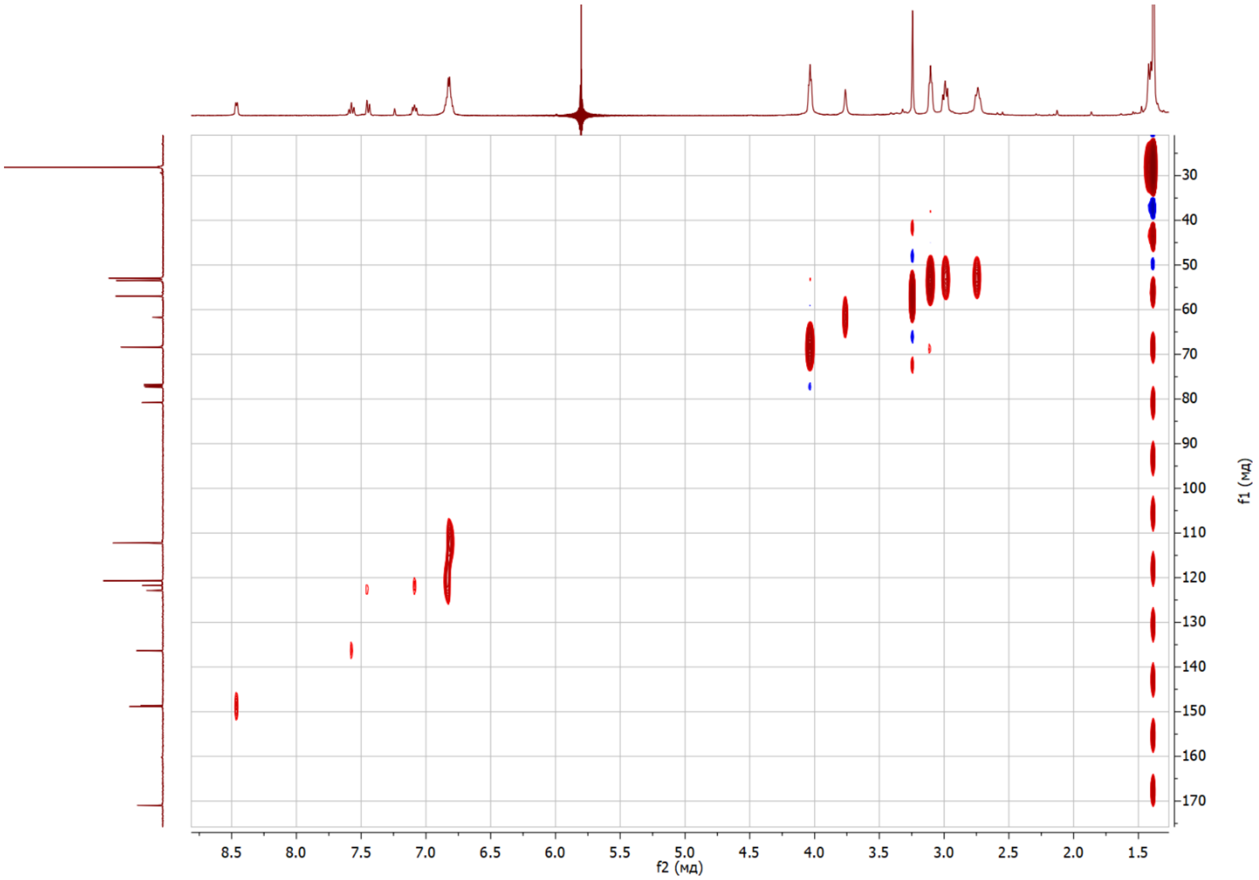

Figure S10. HSQC spectrum of **4** in $CDCl_3$.

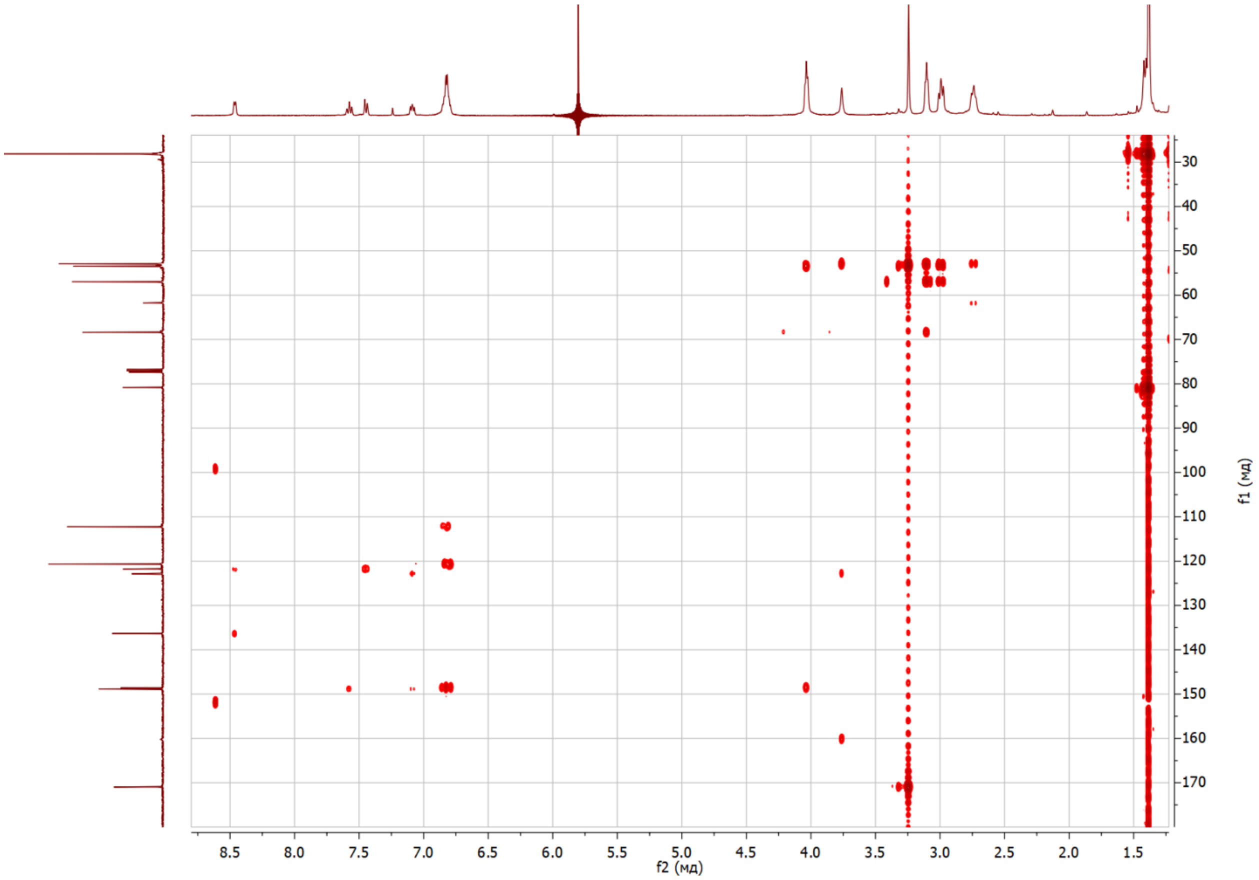

Figure S11. HMBC spectrum of **4** in $CDCl_3$.

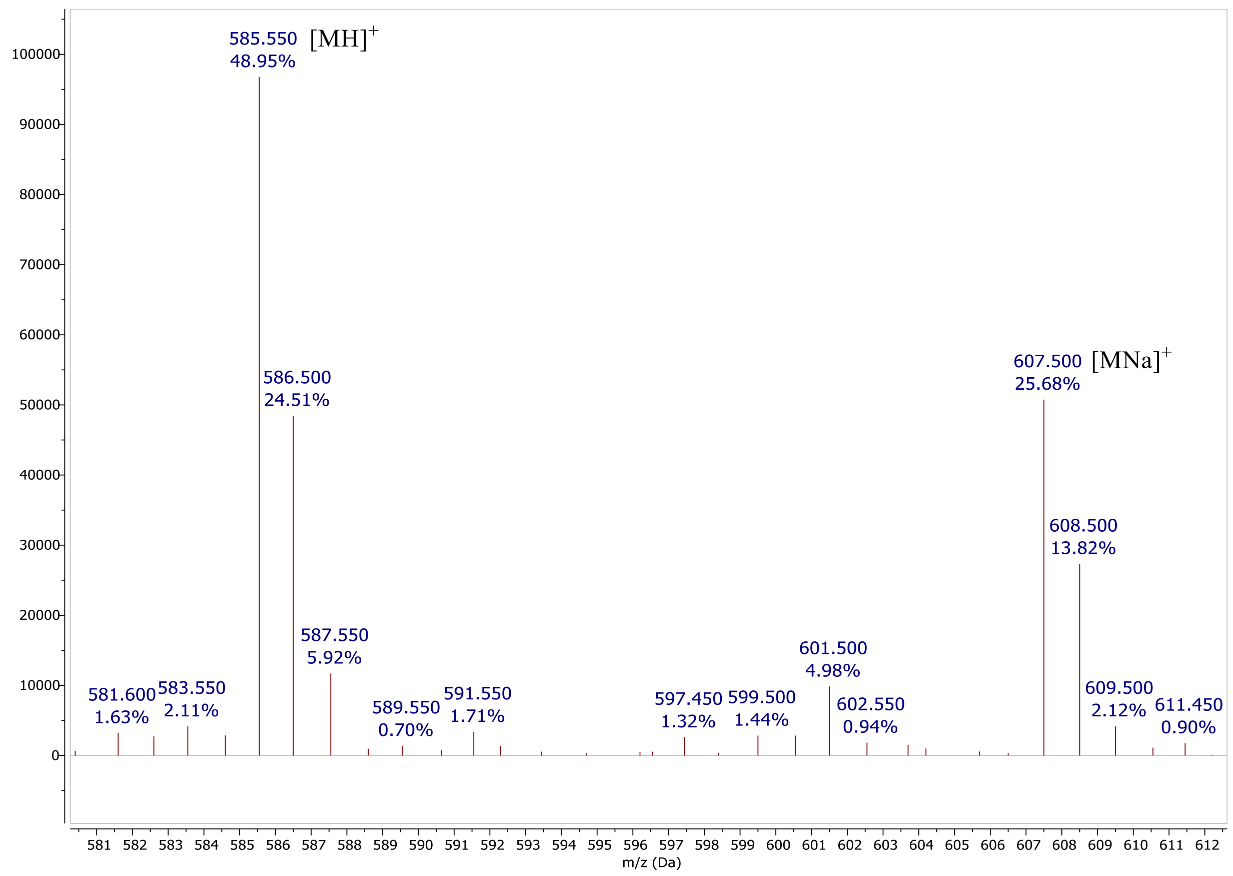

Figure S12. MS (ESI) spectrum of **4**.

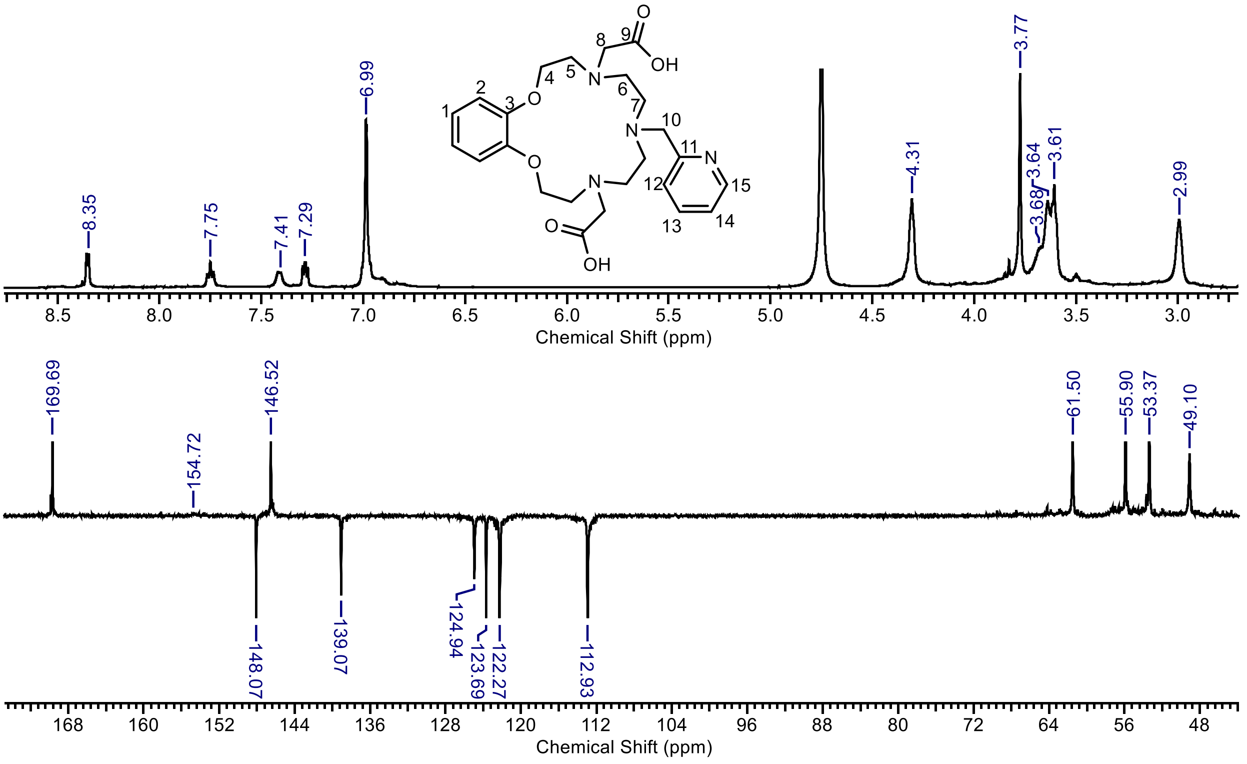

Figure S13. ^{1}H and ^{13}C NMR spectra of **H_2BA2A1Py** in D_2O (pD = 5.7).

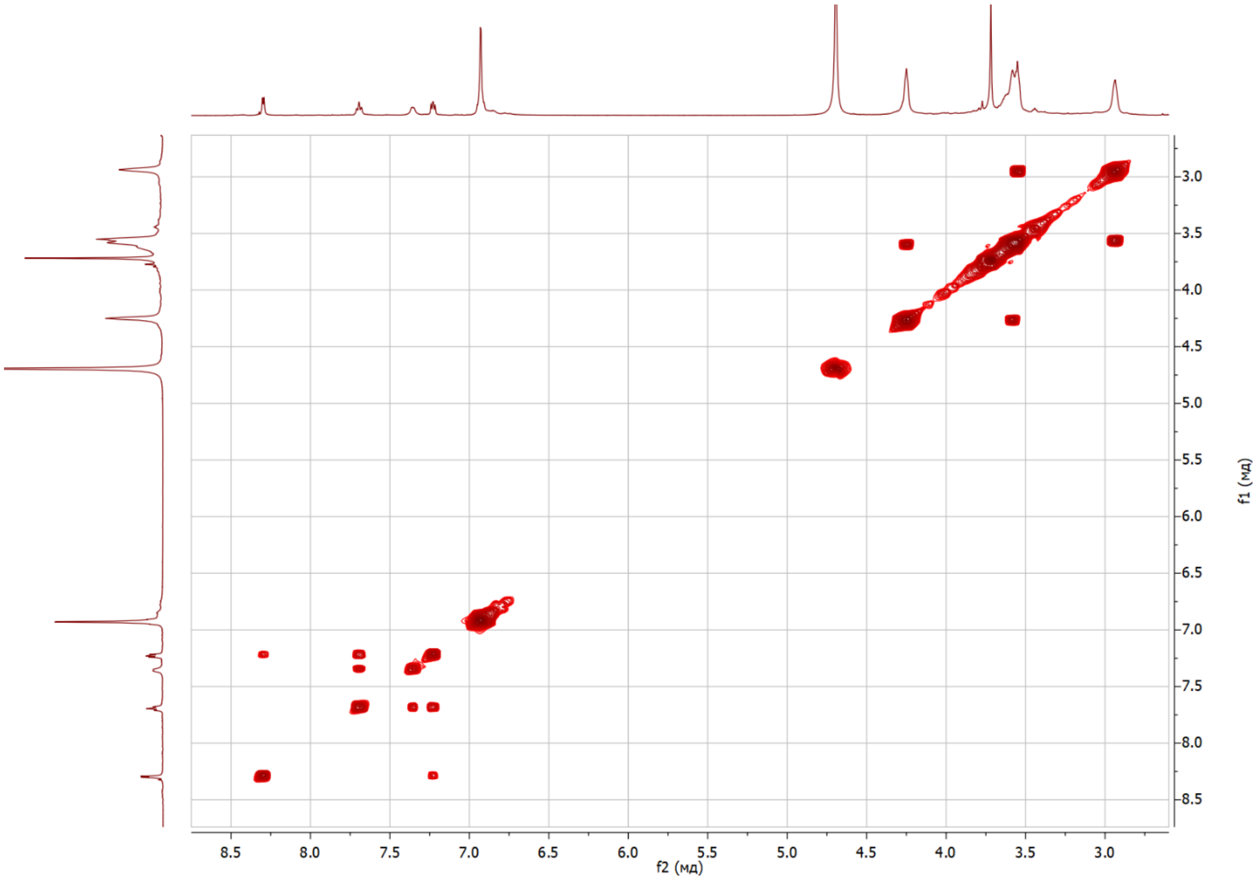

Figure S14. COSY spectrum of **H2BA2A1Py** in D_2O (pD = 5.7).

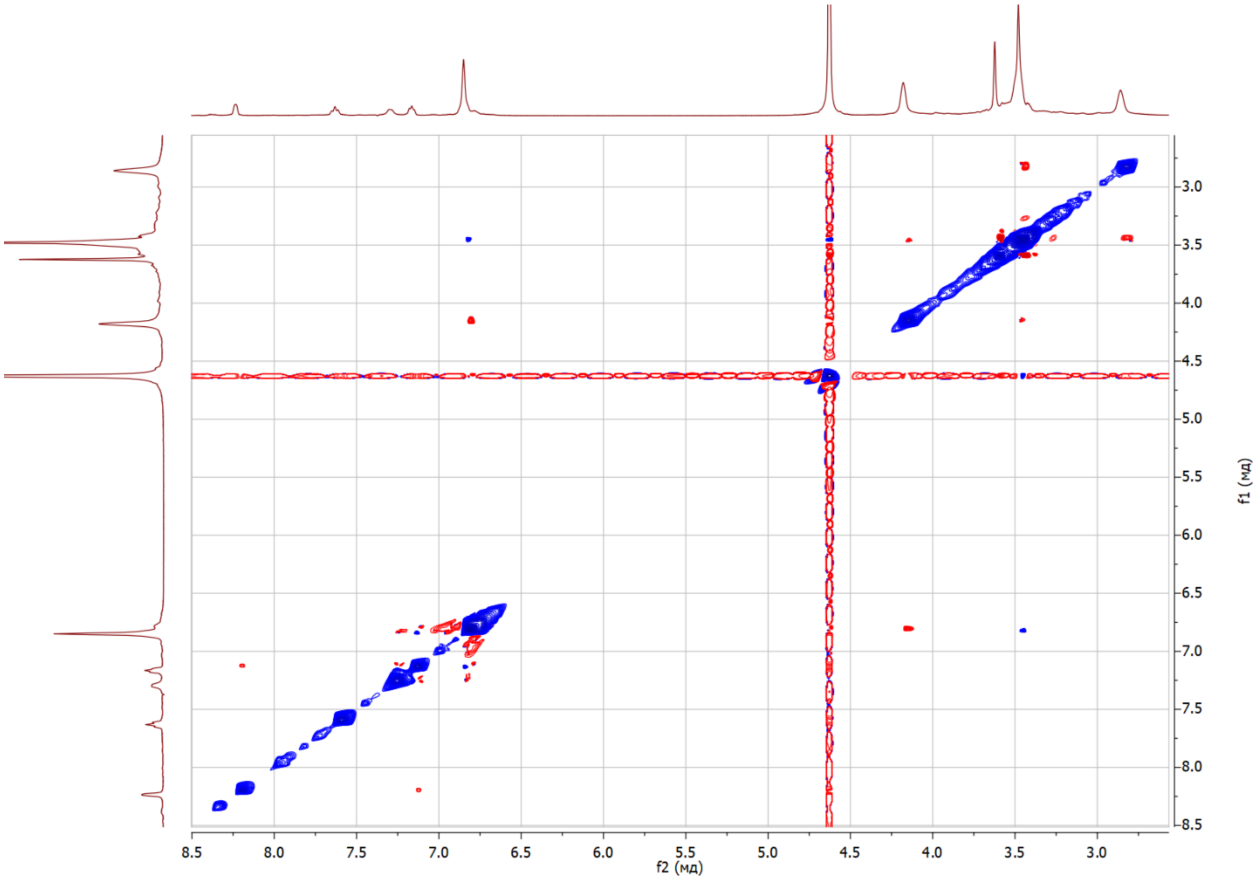

Figure S15. NOESY spectrum of **H2BA2A1Py** in D_2O (pD = 5.7).

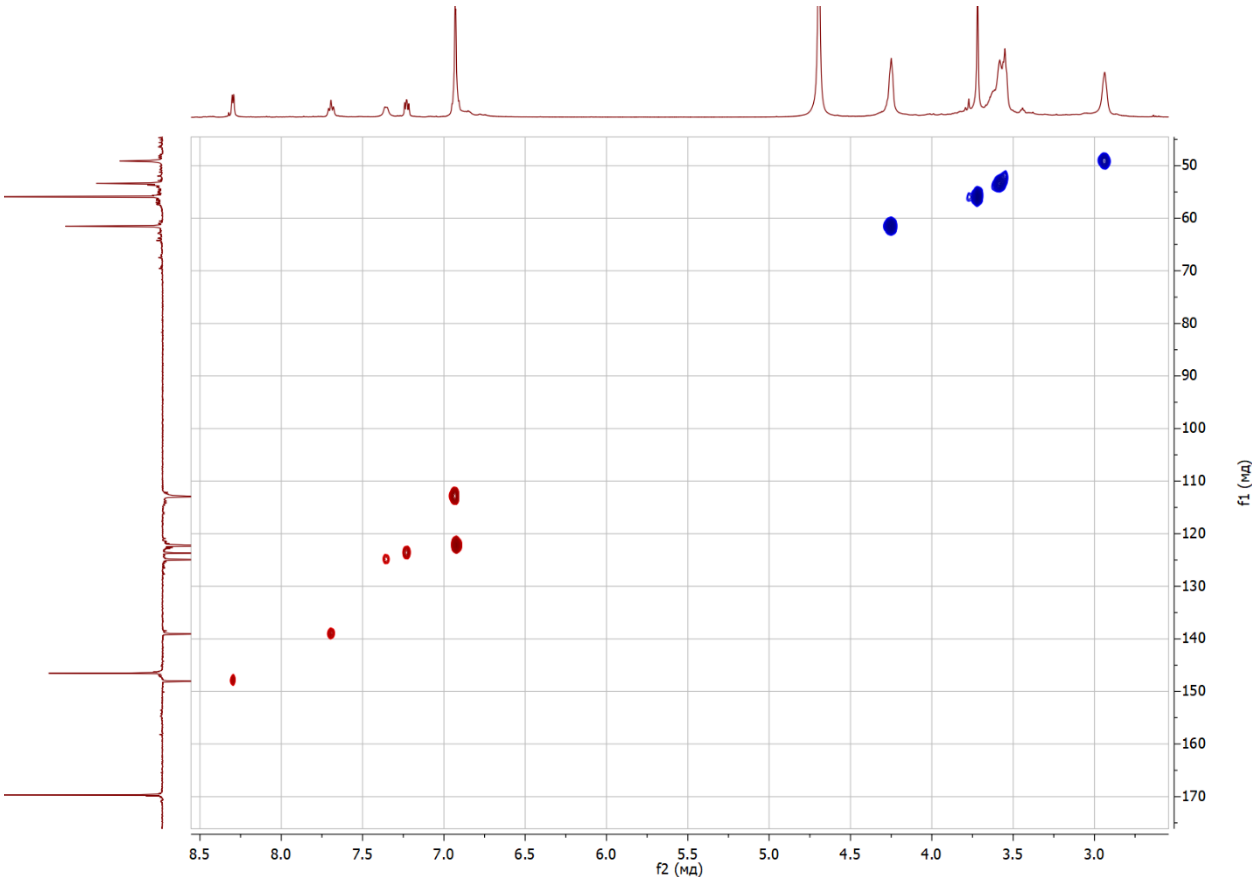

Figure S16. HSQC spectrum of **H_2BA2A1Py** in D_2O (pD = 5.7).

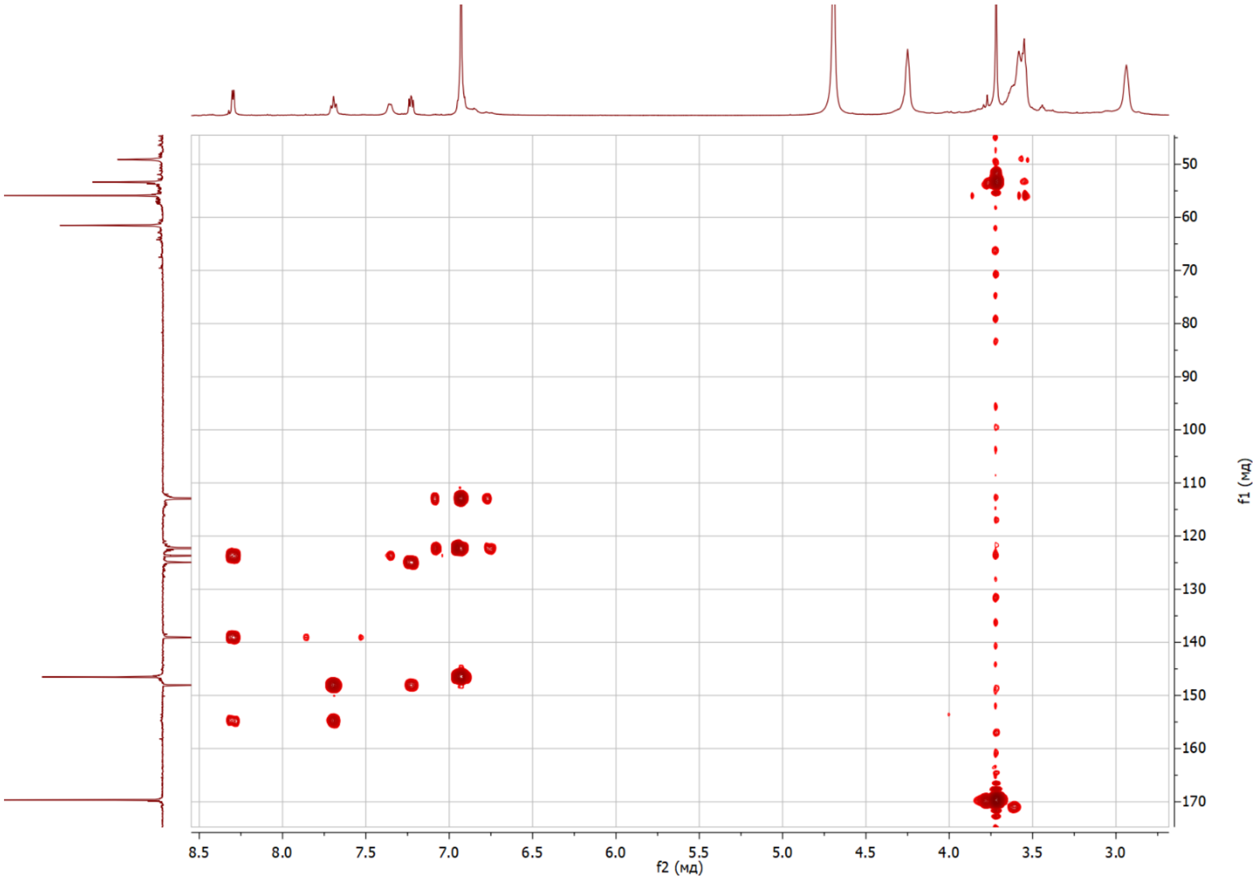

Figure S17. HMBC spectrum of **H_2BA2A1Py** in D_2O (pD = 5.7).

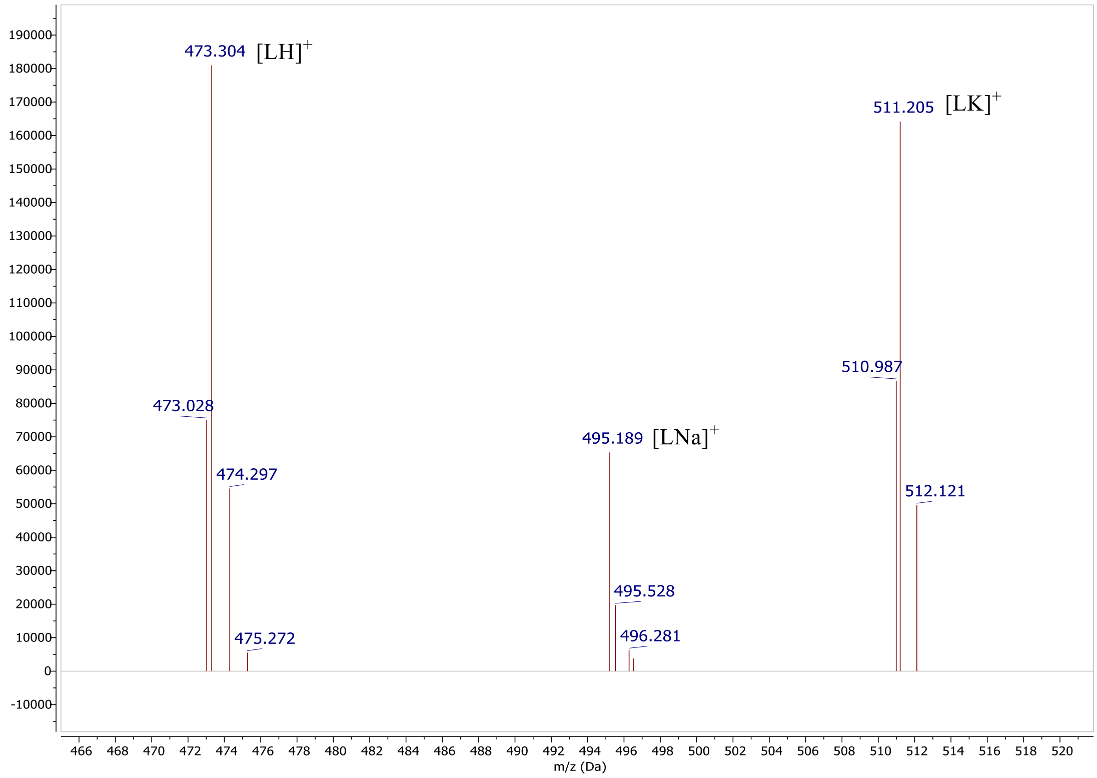

Figure S18. MS (ESI) spectrum of **H_2BA2A1Py.**

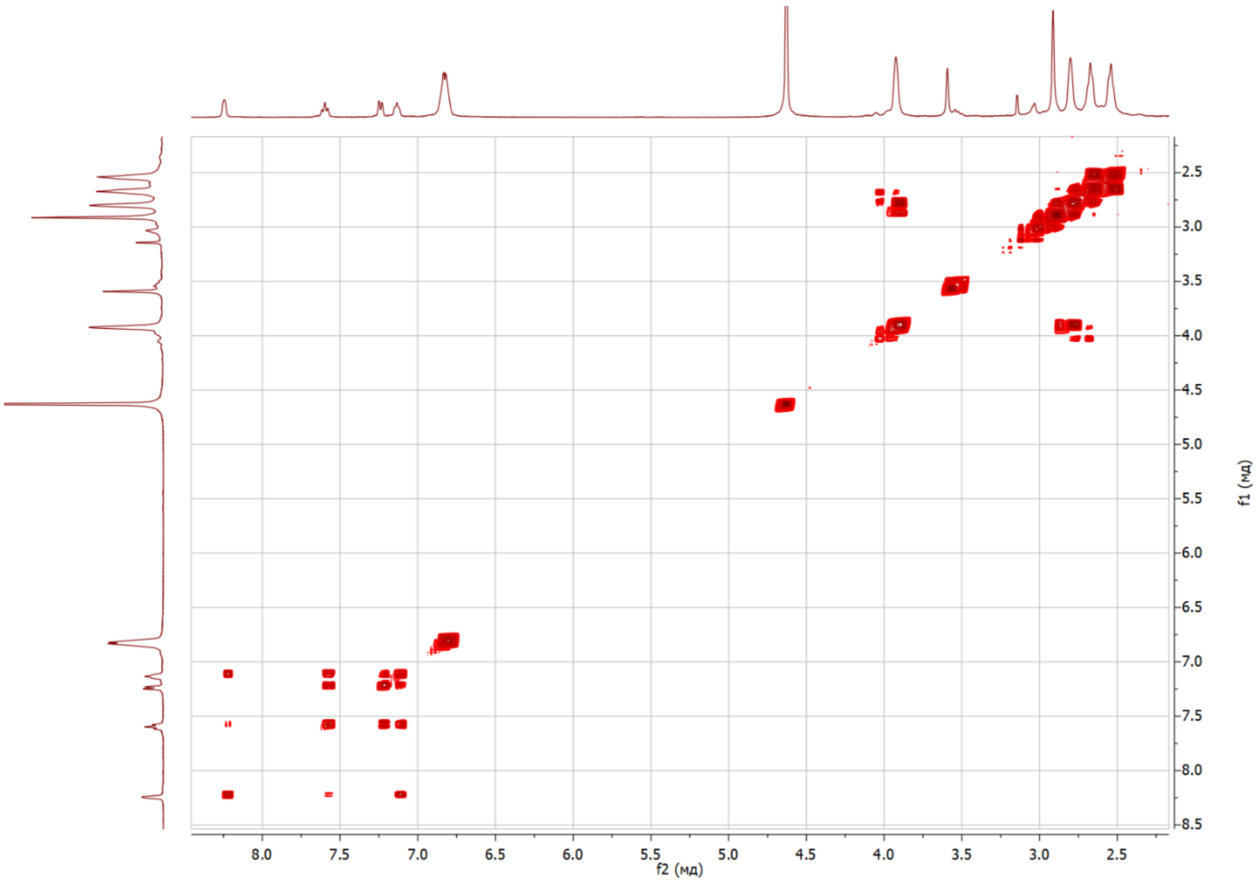

Figure S19. COSY spectrum of **BA2A1Py**$^{2-}$ in D_2O (pD = 11.2).

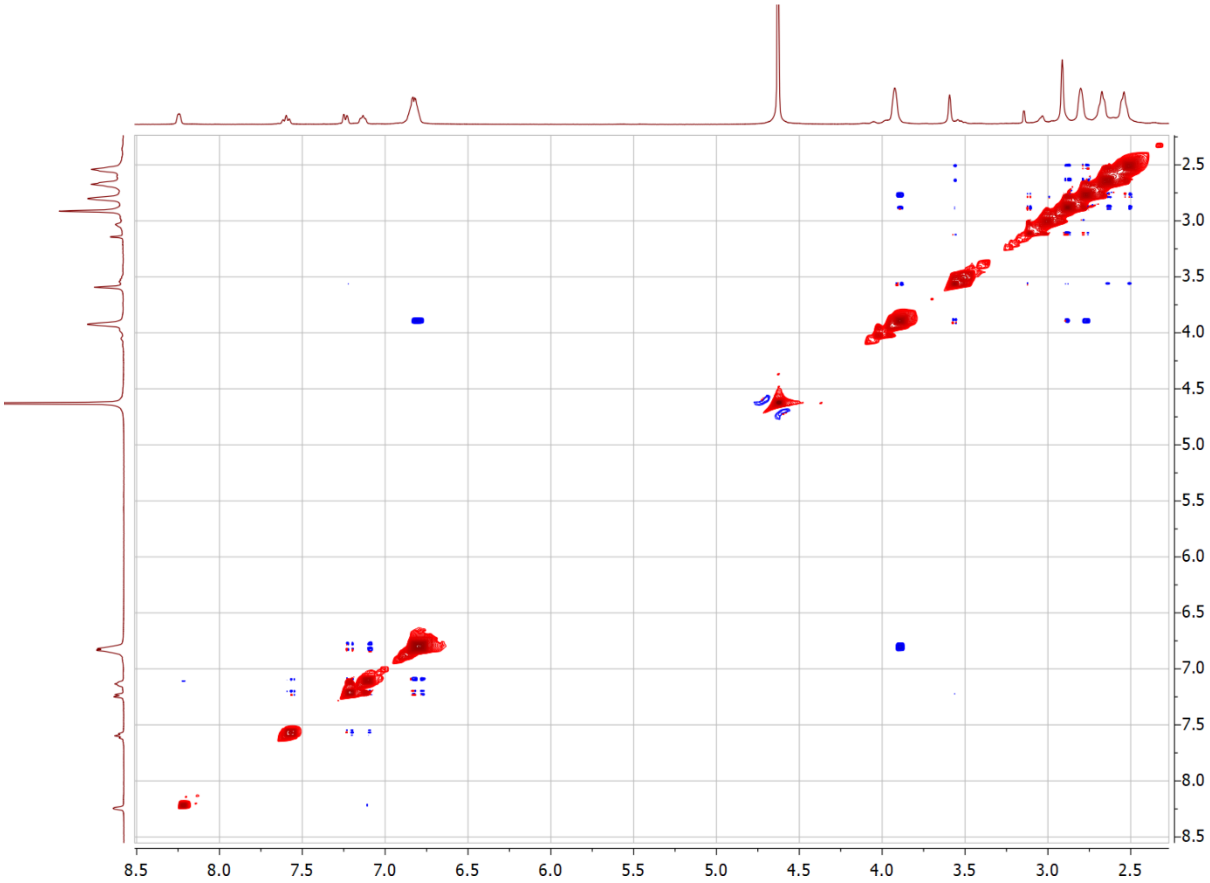

Figure S20. NOESY spectrum of **BA2A1Py**$^{2-}$ in D_2O (pD = 11.2).

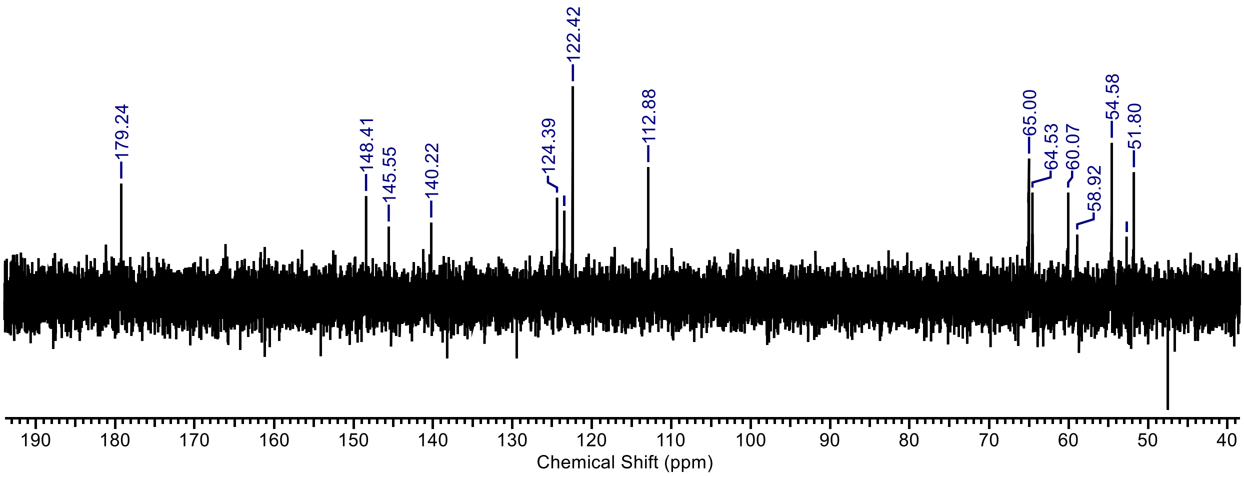

Figure S21. ^{13}C NMR spectrum of **H_2BA2A1Py** in the presence of Pb^{2+} (*C*(L) = 10 мМ, p*D*=6.3) in D_2O.

Table S1. ^{1}H NMR chemical shifts (Δδ, ppm) of **H_2BA2A1Py** recorded in D_2O solution in the absence and presence of Pb^{2+}.

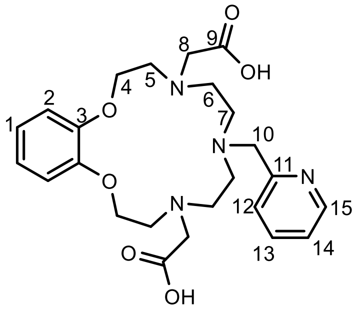

	**BA2A1Py^{2-}** (pD = 11.2)	**HBA2A1Py^{-}** (pD = 8.5)	**H_2BA2A1Py** (pD = 5.7)	**H_3BA2A1Py^{+}** (pD = 2.6)	Pb·**BA2A1Py** (pD = 6.3)	Pb·**BA2A1PyOH^{-}** (pD = 10.5)	Pb·**HBA2A1Py^{+}** (pD = 2.4)
H_1	6.93	6.97 (Δδ = 0.04)	6.99 (Δδ = 0.06)	7.05 (Δδ = 0.12)	7.06 (Δδ = 0.13)	7.06 (Δδ = 0.13)	7.06 (Δδ = 0.13)
H_2							
H_{4a}	4.03	4.18 (Δδ = 0.15)	4.31 (Δδ = 0.28)	4.40 (Δδ = 0.37)	4.31 (Δδ = 0.28)	4.31 (Δδ = 0.28)	4.32 (Δδ = 0.29)
H_{4e}					4.03 (Δδ = 0)	4.02 (Δδ = -0.01)	4.06 (Δδ = 0.03)
H_{5a}	2.91	3.26 (Δδ = 0.35)	3.64 (Δδ = 0.73)	3.70 (Δδ = 0.79)	2.73 (Δδ = -0.18)	2.71 (Δδ = -0.20)	2.80 (Δδ = -0.11)
H_{5e}					3.23 (Δδ = 0.32)	3.20 (Δδ = 0.29)	3.22 (Δδ = 0.31)
H_{6a}	2.78	3.26 (Δδ = 0.48)	3.61 (Δδ = 0.83)	3.68 (Δδ = 0.90)	3.11 (Δδ = 0.33)	3.10 (Δδ = 0.32)	3.13 (Δδ = 0.35)
H_{6e}					2.70 (Δδ = -0.08)	2.71 (Δδ = -0.07)	2.77 (Δδ = -0.01)
H_{7a}	2.64	3.03 (Δδ = 0.39)	2.99 (Δδ = 0.35)	3.09 (Δδ = 0.45)	3.05 (Δδ = 0.41)	3.04 (Δδ = 0.40)	3.09 (Δδ = 0.45)
H_{7e}					2.54 (Δδ = -0.10)	2.54 (Δδ = -0.10)	2.63 (Δδ = -0.01)
H_{8x}	3.02	3.36 (Δδ = 0.34)	3.77 (Δδ = 0.75)	3.83 (Δδ = 0.81)	3.70 (Δδ = 0.68)	3.69 (Δδ = 0.67)	3.74 (Δδ = 0.72)
H_{8y}					2.27 (Δδ = -0.75)	2.25 (Δδ = -0.77)	2.44 (Δδ = -0.58)
H_{10}	3.70	3.92 (Δδ = 0.22)	3.68 (Δδ = -0.02)	4.12 (Δδ = 0.42)	4.57 (Δδ = 0.87)	4.56 (Δδ = 0.86)	4.60 (Δδ = 0.90)
H_{12}	7.34	7.41 (Δδ = 0.07)	7.41 (Δδ = 0.07)	8.05 (Δδ = 0.71)	7.53 (Δδ = 0.19)	7.53 (Δδ = 0.19)	7.56 (Δδ = 0.22)
H_{13}	7.71	7.70 (Δδ = -0.01)	7.75 (Δδ = 0.04)	8.44 (Δδ = 0.73)	7.97 (Δδ = 0.26)	7.97 (Δδ = 0.26)	8.00 (Δδ = 0.29)
H_{14}	7.24	7.28 (Δδ = 0.04)	7.29 (Δδ = 0.05)	7.90 (Δδ = 0.66)	7.47 (Δδ = 0.23)	7.47 (Δδ = 0.23)	7.51 (Δδ = 0.27)
H_{15}	8.35	8.36 (Δδ = 0.01)	8.35 (Δδ = 0)	8.62 (Δδ = 0.27)	8.38 (Δδ = 0.03)	8.33 (Δδ = -0.02)	8.41 (Δδ = 0.06)

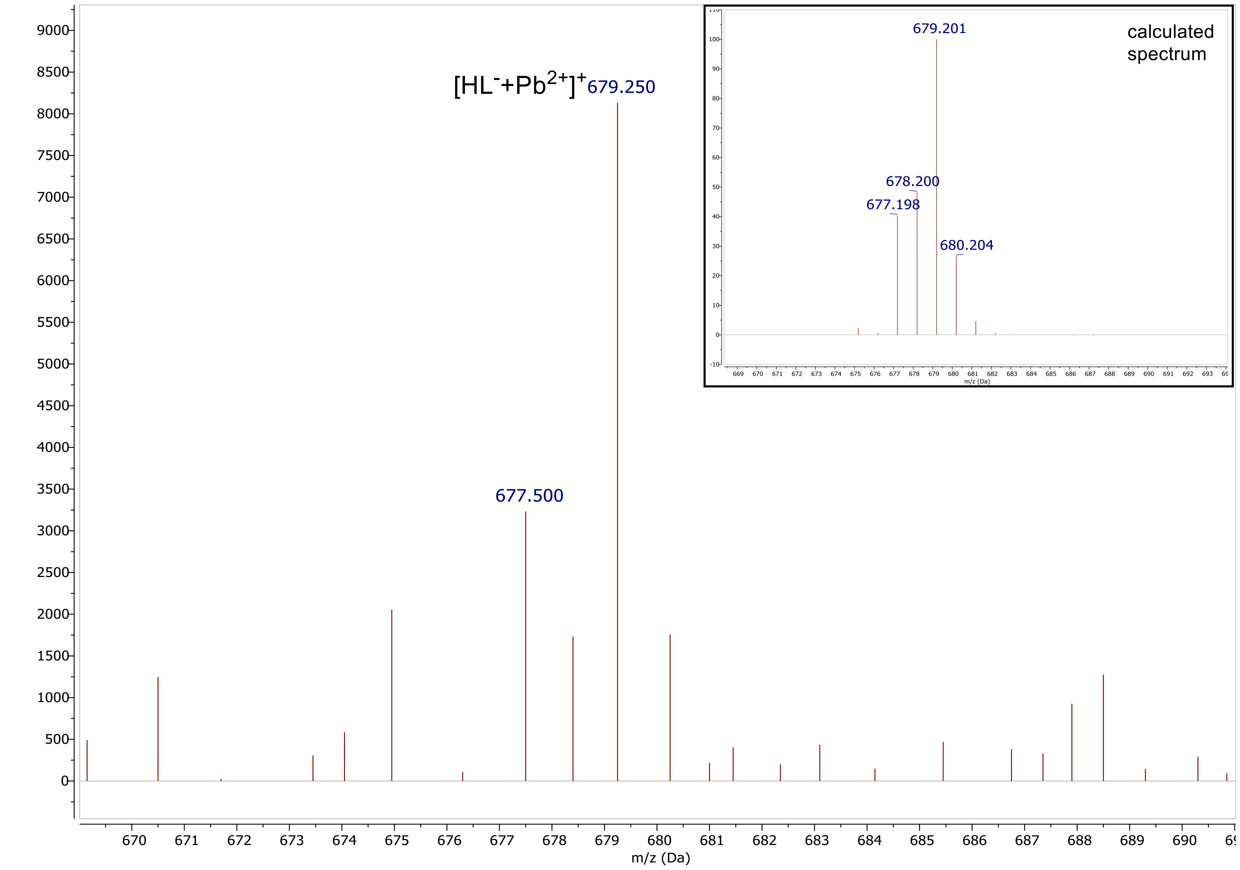

Figure S22. MS (ESI) spectrum of H_2BA2A1Py in the presence of Pb^{2+} in water.

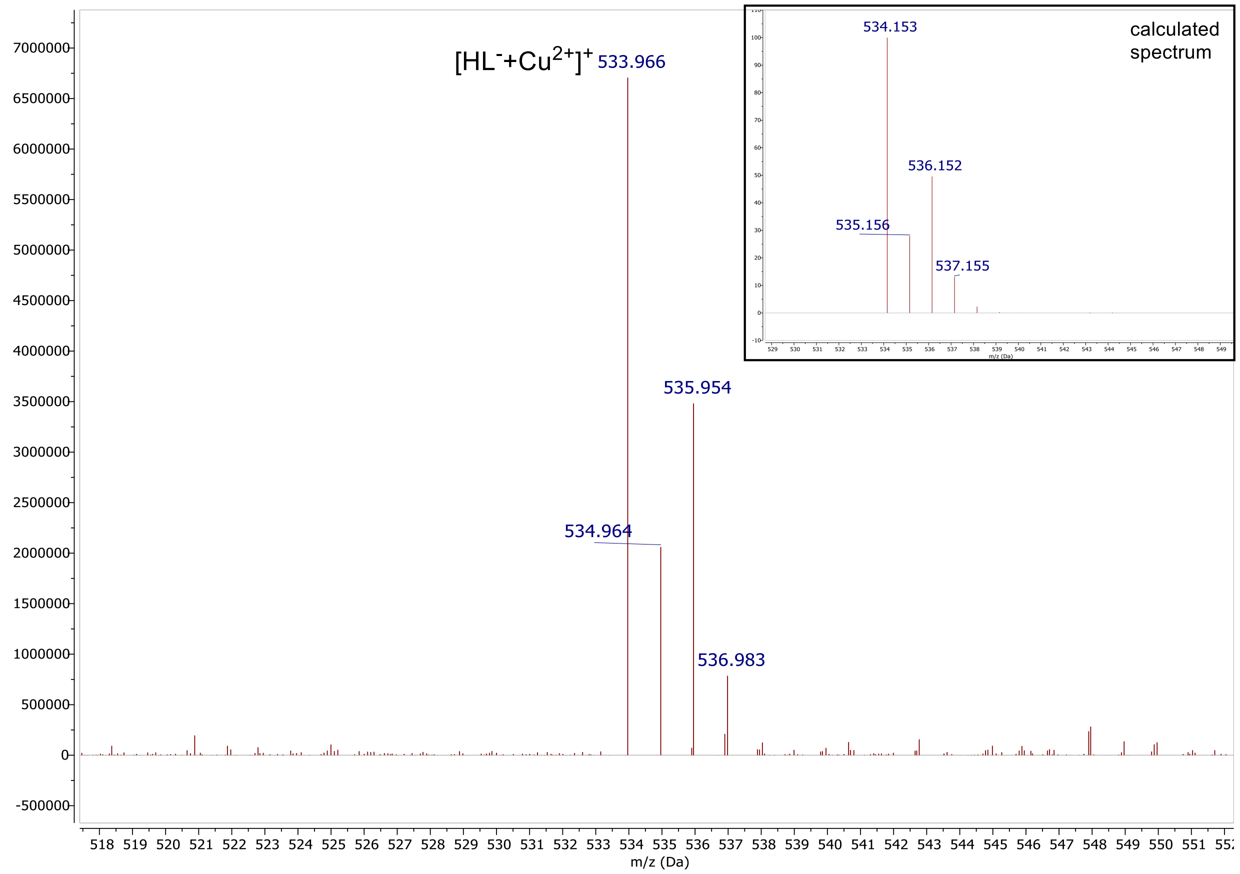

Figure S23. MS (ESI) spectrum of of H_2BA2A1Py in the presence of Cu^{2+} in water.

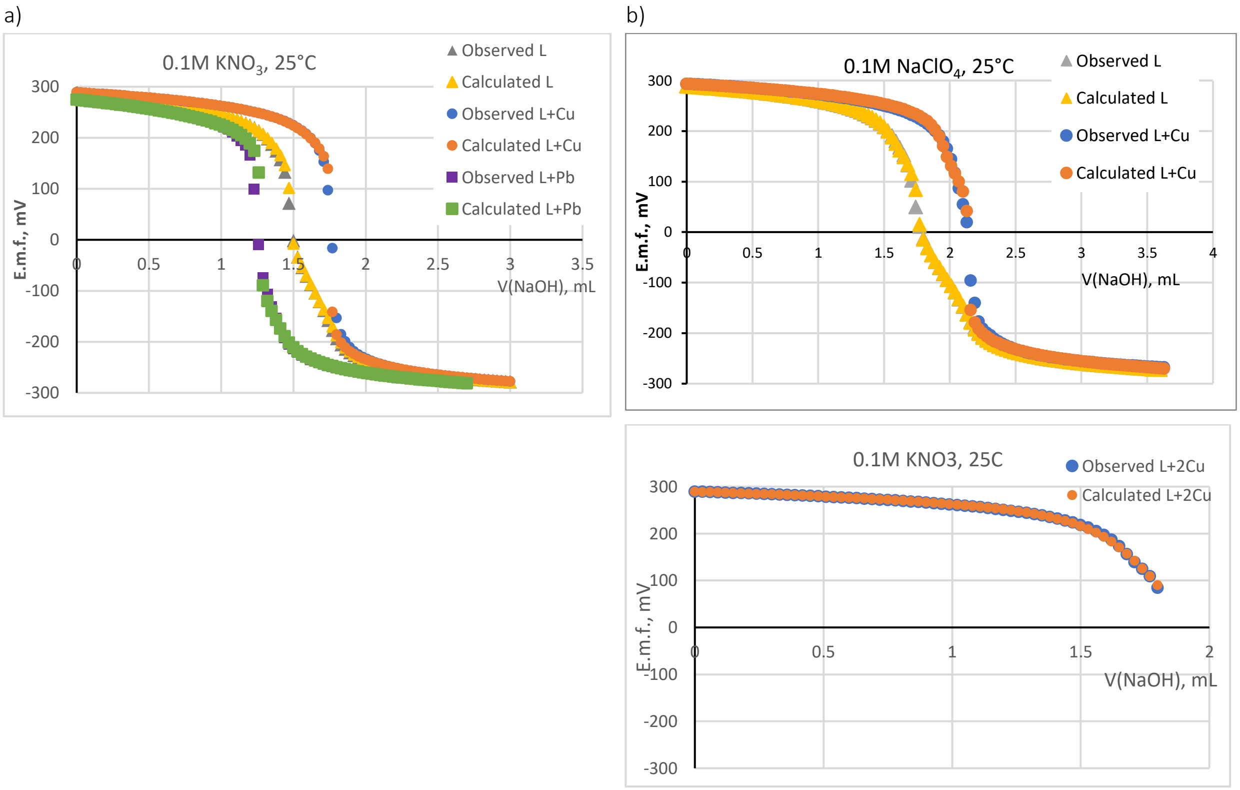

Figure S24. Potentiometric titration curves of solutions, containing 1 mM of L and 1 mM of L with 1 mM of M^{2+} in 0.1 M KNO_3 (a) 0.1M $NaClO_4$ (b) and 1 mM of L with 2 mM of Cu^{2+} in 0.1 M KNO_3.

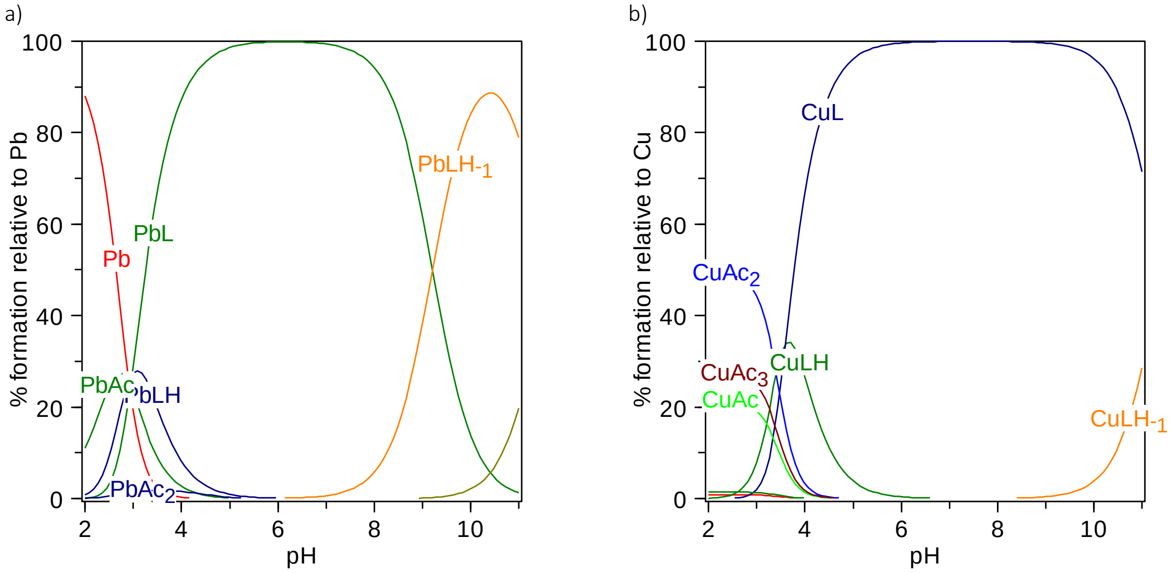

Figure S25. Species distribution diagrams in the systems: a) Pb^{2+} (10 nM), $H_2BA2A1Py$ (0.1mM), Ac^-(0.15 M); b) Cu^{2+} (10 nM), $H_2BA2A1Py$ (0.1mM), Ac^-(0.15 M).

a)

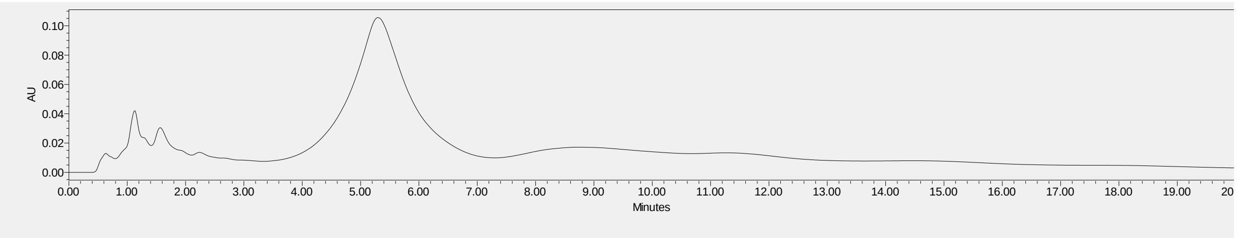

b)

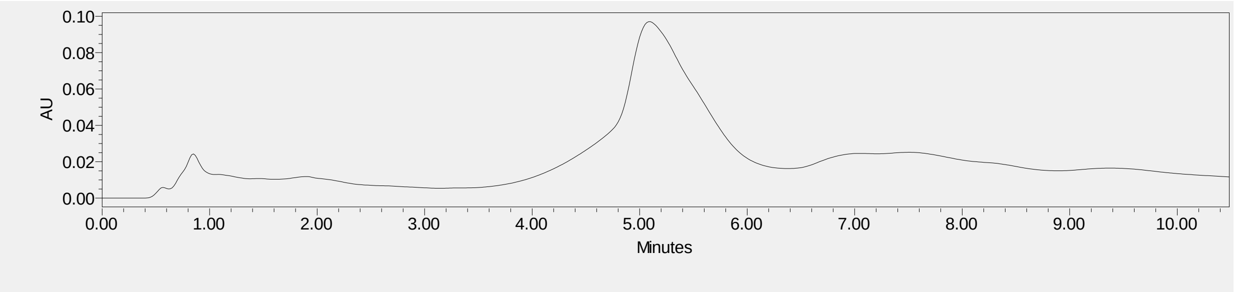

c)

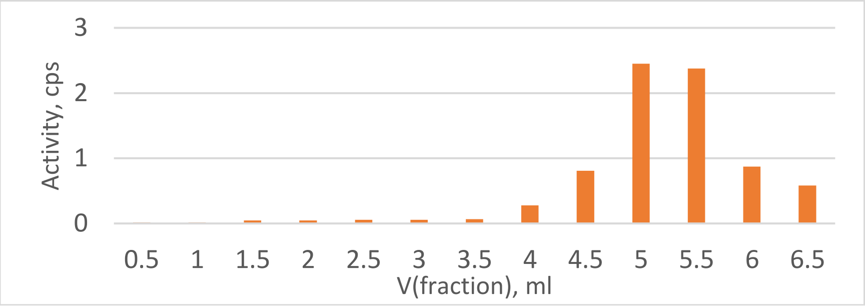

Figure S26. Chromatograms of: a) Cu-BA2A1Py (non-radioactive, 1.7 mM, pH5.3) recorded by UV-Vis detector at 267 nm; b) Pb-BA2A1Py (non-radioactive, 2 mM, pH7.4) recorded by UV-Vis detector at 267 nm; c) [^{210}Pb]Pb-BA2A1Py (radioactive, c(L) = 0.1mM, pH5.3, without NaOAc) plotted according to measured activity in the collected fractions (correction to dead volume was applied).

a)

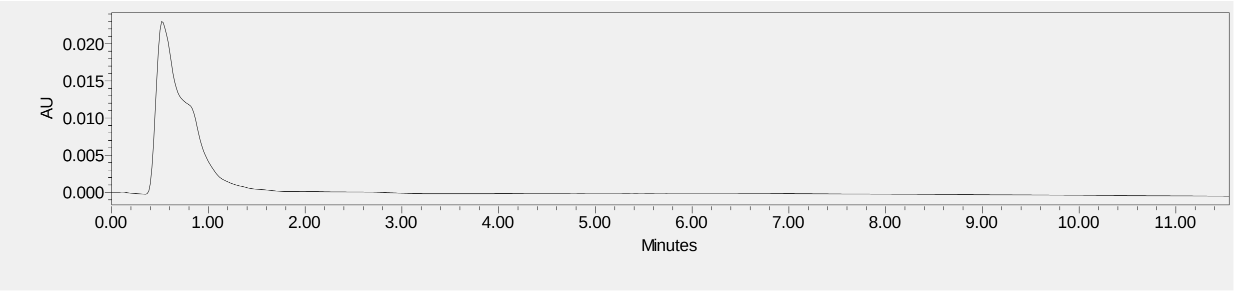

b)

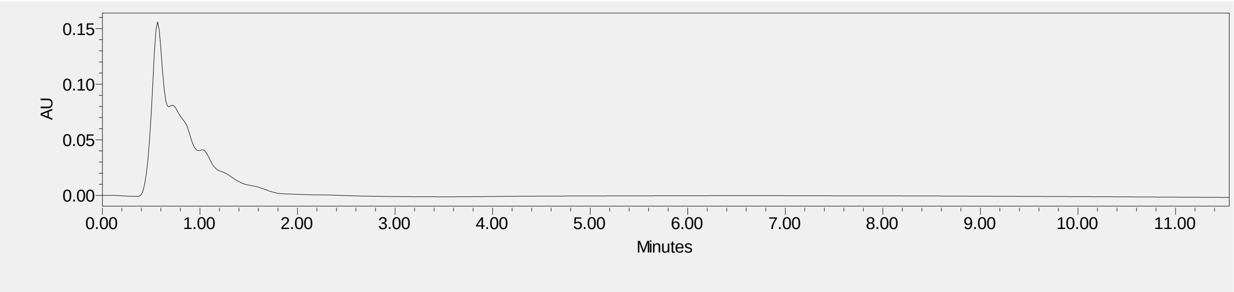

Figure S27. Chromatogram of AcONa (0.15M) recorded by UV-Vis detector at 221 nm (a) and 267 nm (b), in the mode used for Pb-BA2A1Py complex (isocratic, H_2O – 0.9, CH_3CN – 0.1).
